# Genome-Wide Identification, Characterization and Phylogenetic Analysis of ATP-Binding Cassette (ABC) Transporter Genes in Common Carp (*Cyprinus carpio*)

**DOI:** 10.1371/journal.pone.0153246

**Published:** 2016-04-08

**Authors:** Xiang Liu, Shangqi Li, Wenzhu Peng, Shuaisheng Feng, Jianxin Feng, Shahid Mahboob, Khalid A. Al-Ghanim, Peng Xu

**Affiliations:** 1 CAFS Key Laboratory of Aquatic Genomics and Beijing Key Laboratory of Fishery Biotechnology, Centre for Applied Aquatic Genomics, Chinese Academy of Fishery Sciences, Beijing, China; 2 Department of Aquaculture, College of Animal Sciences, Shanxi Agriculture University, Taigu, Shanxi, China; 3 Henan Academy of Fishery Sciences, Zhengzhou, China; 4 Department of Zoology, College of Science, King Saud University, Riyadh, Saudi Arabia; 5 Department of Zoology, GC University, Faisalabad, Pakistan; 6 College of Ocean & Earth Science, Xiamen University, Xiamen, China; University of Technology Sydney, AUSTRALIA

## Abstract

The ATP-binding cassette (ABC) gene family is considered to be one of the largest gene families in all forms of prokaryotic and eukaryotic life. Although the ABC transporter genes have been annotated in some species, detailed information about the ABC superfamily and the evolutionary characterization of ABC genes in common carp (*Cyprinus carpio*) are still unclear. In this research, we identified 61 ABC transporter genes in the common carp genome. Phylogenetic analysis revealed that they could be classified into seven subfamilies, namely 11 ABCAs, six ABCBs, 19 ABCCs, eight ABCDs, two ABCEs, four ABCFs, and 11 ABCGs. Comparative analysis of the ABC genes in seven vertebrate species including common carp, showed that at least 10 common carp genes were retained from the third round of whole genome duplication, while 12 duplicated ABC genes may have come from the fourth round of whole genome duplication. Gene losses were also observed for 14 ABC genes. Expression profiles of the 61 ABC genes in six common carp tissues (brain, heart, spleen, kidney, intestine, and gill) revealed extensive functional divergence among the ABC genes. Different copies of some genes had tissue-specific expression patterns, which may indicate some gene function specialization. This study provides essential genomic resources for future studies in common carp.

## Introduction

The ATP-binding cassette (ABC) transporters are integral membrane proteins and are one of the largest superfamilies ubiquitously present in all phyla [1]. The majority of ABC proteins function as primary active transporters [2], requiring the binding and hydrolysis of ATP to transport numerous substrates (e.g., simple ions, amino acids, lipids, sugars, peptides, and drugs) from the cytosol to intracellular or extracellular regions [3]. Typical ABC proteins possess two integral transmembrane domains (TMDs) and two cytosolic nucleotide-binding domains (NBDs) that bind and hydrolyze ATP [4]. ABC transporters that contain only one NBD and one TMD need to form homo- or heterodimers to generate a functional pump [5,6]. Based on sequence similarity among the NBDs, the ABC superfamily has been divided into eight subfamilies, named by the letters A–H [3].

ABC transporters were first described in bacteria in the 1970s as substrate-binding protein-dependent transport systems. In 1986, it was recognized that the ATP-binding subunits shared a common evolution origin, although the term ABC transporter was not used until 1990 [7]. The ABC transporter family was first characterized in human with a total of 48 members among animals [8]. In human, mutations in many ABC genes have been associated with hereditary diseases, including cystic fibrosis [9], adrenoleukodystrophy [10], and cholesterol metabolism disorders [11]. ABC proteins that function as drug transporters can contribute to chemical resistance phenotypes and reduce the susceptibility of cancer cells, which has been defined as multidrug resistance (MDR) in tumors [12]. Multidrug transporters that can cause MDR are members of subfamilies B, C, and G and include ABCB1 (also known as MDR1 or P-glycoprotein), ABCC1 (also known as MRP1) and ABCG2 (also known as BCRP) [13]. ABC transporters have also been linked to drug resistance in parasitic nematodes and to pesticide resistance in insects and other arthropods [2].

Comparing with that in mammals, the characterization of teleost ABC transporters is more complicated, mainly because of the greater pressures suffered by teleost fishes in their aquatic environment. Due to ABC transporters have been associated with biochemical defense against environmental toxins, extensive studies have been conducted on teleost ABC transporters. For instance, Liu et al. reported that ABC transporters may participate in detoxification pathways in catfish [3], and Jeong et al. revealed ABCH nomenclature in medaka [14]. The lack of genomic resources is one of the obstacles to characterizing ABC transporter families in teleost, and many studies have been limited mainly to model species such as medaka and zebrafish.

Common carp (*Cyprinus carpio*), one of the most significant teleost species, is widespread all over the world and especially in Europe and Asia. Numerous genomic resources for common carp are now available, including a large number of expressed sequence tags (ESTs) [15], bacterial artificial chromosome (BAC)-end sequences [16], comprehensive transcriptome sequences obtained by RNA-seq [17,18], single nucleotide polymorphisms (SNPs) [19], and genetic and physical maps [20,21]. The common carp genome has recently been completely sequenced and the predicted genes have been annotated [22]. Owing to that the common carp genome had experienced additional rounds of whole genome duplication (WGD) comparing with the genomes of most other teleost, the complexity of the allotetraploid genome and gene duplications will provide an important model for exploring gene fates in newly duplicated genomes. Here we focus on the ABC gene families in common carp and perform phylogenic and orthologous analyses, to obtain valuable clues and evidences about gene evolution and fates post WGD.

## Results and Discussion

### Identification and phylogenetic analysis of ABC transporter genes in common carp

We identified a total of 61 ABC transporter genes in the common carp genome that we classified as follows: 11 ABCAs, six ABCBs, 19 ABCCs, eight ABCDs, two ABCEs, four ABCFs, and 11 ABCGs. The transcripts, coding sequences, and locations of these ABCs are summarized in Table 1. All sequences are available in S1 Table.

**Table 1:** The ABC transporter gene subfamilies in the common carp genome.

Gene name	Genomic length (bp)	CDS (na)^a^	CDS (aa)^b^	CDS status	Location
**ABCA1a-1**	12683	3138	1046	partial	scaffold
**ABCA1a-2**	20508	3120	1039	partial	LG2
**ABCA1b-1**	12339	2946	982	partial	LG28
**ABCA1b-2**	20132	4986	1661	partial	LG28
**ABCA2**	15121	4734	1578	partial	scaffold
**ABCA3b**	18382	2031	676	partial	scaffold
**ABCA4a**	20517	2829	942	partial	LG48
**ABCA4b**	21288	3261	1087	partial	scaffold
**ABCA5-1**	13463	1503	500	partial	LG33
**ABCA5-2**	20907	3318	1106	partial	scaffold
**ABCA12**	21966	3411	1137	partial	LG11
**ABCB4**	27159	2520	839	complete	LG32
**ABCB5-1**	20931	2274	757	complete	LG32
**ABCB5-2**	27476	3279	1092	complete	LG37
**ABCB9**	5885	798	265	complete	LG45
**ABCB11a**	28229	1362	453	complete	LG41
**ABCB11b**	30493	2547	848	complete	scaffold
**ABCC1**	10702	1665	555	partial	scaffold
**ABCC2-1**	26977	3168	1055	complete	scaffold
**ABCC2-2**	27106	3516	1171	complete	scaffold
**ABCC4-1**	33014	2699	1232	partial	scaffold
**ABCC4-2**	23070	2100	699	complete	scaffold
**ABCC5-1**	24438	3318	1106	partial	LG36
**ABCC5-2**	17139	1860	620	partial	scaffold
**ABCC6a**	22516	2865	954	complete	scaffold
**ABCC6-2**	20481	2853	950	complete	LG5
**ABCC6-3**	21709	1935	644	complete	LG47
**ABCC7**	61138	3855	1285	partial	LG36
**ABCC8**	17975	1356	451	partial	scaffold
**ABCC8-like**	16190	1980	660	partial	LG9
**ABCC9-1**	30404	3096	1031	complete	scaffold
**ABCC9-2**	19447	2274	758	partial	scaffold
**ABCC10**	11264	3315	1105	partial	LG21
**ABCC12-1**	9613	2550	850	partial	LG14
**ABCC12-2**	20367	2052	683	partial	LG13
**ABCC13**	28867	2493	830	complete	scaffold
**ABCD1**	23161	2616	871	partial	scaffold
**ABCD2**	22580	2055	684	complete	LG32
**ABCD3a-1**	20678	2514	837	complete	scaffold
**ABCD3a-2**	20436	2841	946	complete	scaffold
**ABCD3b**	20571	1845	614	complete	scaffold
**ABCD4-1**	22007	4767	1589	partial	LG47
**ABCD4-2**	16733	3948	1315	complete	scaffold
**ABCD4-like**	13237	2220	740	partial	LG6
**ABCE1-1**	24420	1281	426	complete	scaffold
**ABCE1-2**	27484	1296	431	complete	scaffold
**ABCF1-like**	25517	2952	983	complete	scaffold
**ABCF2a**	21339	2280	760	partial	scaffold
**ABCF2-2**	21460	3183	1060	partial	LG44
**ABCF3**	21366	1449	482	partial	scaffold
**ABCG1**	20586	1767	588	complete	LG30
**ABCG2-like**	30751	2709	902	complete	LG49
**ABCG2**	14507	1170	389	complete	LG45
**ABCG2b**	5852	1041	346	complete	scaffold
**ABCG2c**	10722	1542	513	complete	scaffold
**ABCG2d**	8650	1335	444	complete	scaffold
**ABCG4**	26239	1947	648	partial	LG10
**ABCG4b**	25490	1791	596	complete	scaffold
**ABCG5**	9348	1191	396	complete	scaffold
**ABCG8-1**	23381	1704	567	complete	scaffold
**ABCG8-2**	5165	705	234	partial	scaffold

^a^ Nucleic acid sequence.

^b^ Amino acid sequence.

#### ABCA subfamily

**Fig 1:**
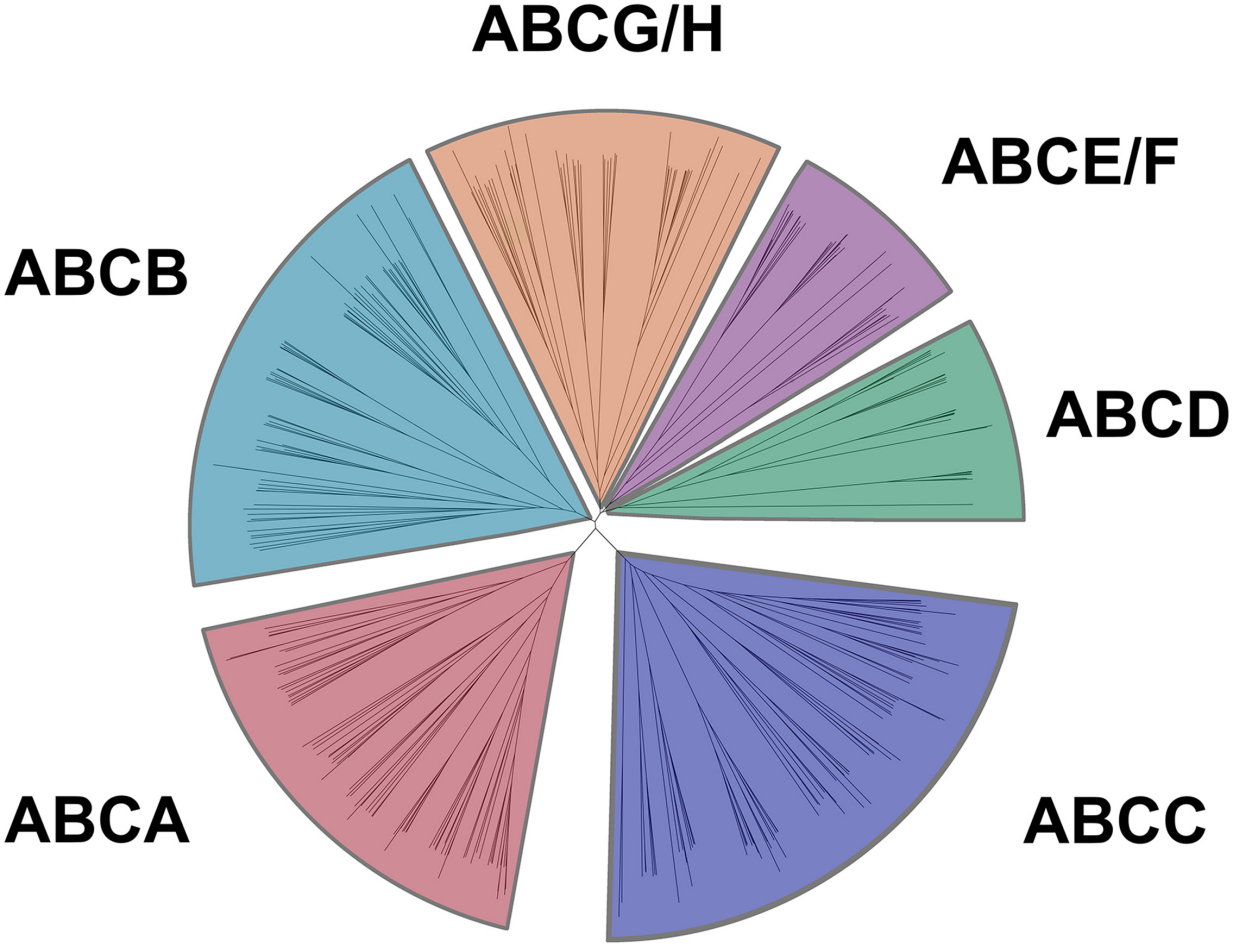
Phylogenetic tree of the ABC transporter family in seven species including common carp. Neighbor-joining-based phylogenetic tree of ABC proteins sequences from seven vertebrate species: human (Hsa), mouse (Mmu), chicken (Gga), frog (Xtr), medaka (Ola), zebrafish (Dre), and common carp (Cca). The ABC subfamilies are labeled from A–H.

**Fig 2:**
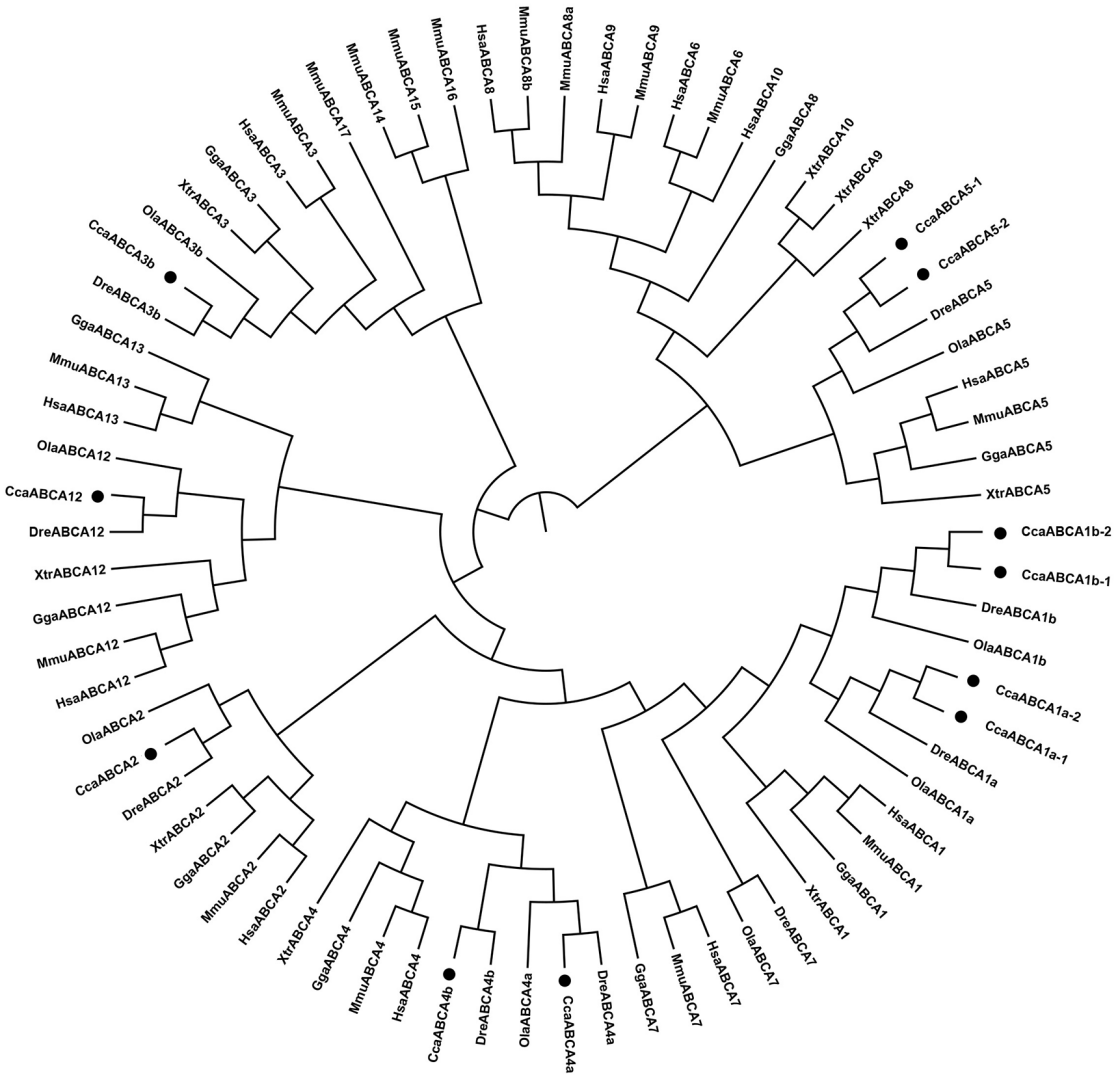
Phylogenetic tree of the ABCA subfamily transporters. Neighbor-joining-based phylogenetic tree of ABCA proteins sequences. Human (Hsa), mouse (Mmu), chicken (Gga), frog (Xtr), medaka (Ola), zebrafish (Dre), and common carp (Cca). The black dots indicate common carp genes.

The eleven ABCA genes identified in the common carp genome were annotated as ABCA1a-1, ABCA1a-2, ABCA1b-1, ABCA1b-2, ABCA2, ABCA3b, ABCA4a, ABCA4b, ABCA5-1, ABCA5-2, and ABCA12 (Table 1). The phylogenetic analysis showed that each ABCA subfamily clustered with the orthologs from the other species (Figs 1 and 2). We identified four copies of ABCA1 (ABCA1a-1, ABCA1a-2, ABCA1b-1, and ABCA1b-2) in common carp, while only two copies have been identified in zebrafish and medaka. We also identified two copies of ABCA4 (ABCA4a and ABCA4b) and ABCA5 (ABCA5-1 and ABCA5-2), and only single copy of ABCA2, ABCA3b, and ABCA12. In the ABCA5 clade, the common carp ABCA5s were grouped with the other ABCA5s, and this group then clustered with ABCA6/8/9/10 sequences from the tetrapods, even though orthologous sequences were not detected in the common carp genome and have not been reported in other teleosts such as zebrafish and medaka. This finding implies that ABCA5-related gene divergence may have occurred in tetrapods [23]. We found a similar scenario in the ABCA3 clade, where ABCA14/15/16/17 were expanded in mouse. Unexpectedly, no ABCA7 gene was detected in the common carp genome, even though one ABCA7 copy is retained in both the zebrafish and medaka genomes. Further investigations are necessary to verify whether ABCA7 was lost during evolution or it was missed because of inaccurate gene prediction or annotation.

#### ABCB subfamily

**Fig 3:**
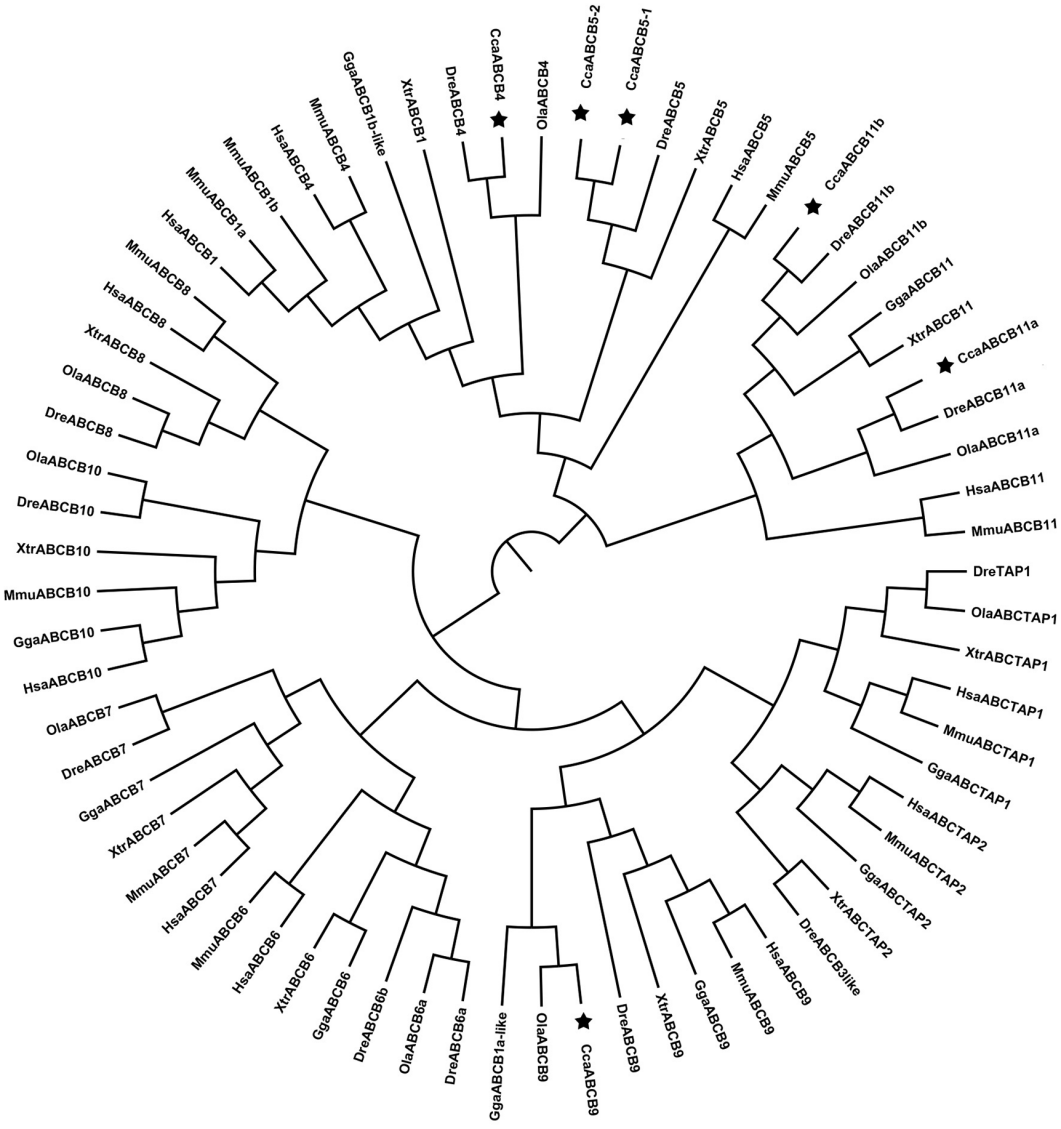
Phylogenetic tree of ABCB subfamily transporters. Neighbor-joining-based phylogenetic tree of ABCB proteins sequences. Human (Hsa), mouse (Mmu), chicken (Gga), frog (Xtr), medaka (Ola), zebrafish (Dre), and common carp (Cca). The black stars indicate common carp genes.

We detected only six ABCB genes in the common carp genome compared with twelve in zebrafish and nine in medaka. The common carp ABCB genes were annotated as ABCB4, ABCB5-1, ABCB5-2, ABCB9, ABCB11a, and ABCB11b (Table 1). The phylogenetic analysis showed that the full-length coding sequences were obtained for the six ABCB transporters (Table 1) and all the common carp ABCB transporters clustered with the orthologs from the other species (Figs 1 and 3). The ABCBs clustered into two distinct clades with ABCB1, ABCB4, ABCB5, and ABCB11 in one clade, and ABCB8/10/7/6/9/tap1/tap2 in another clade. The phylogenetic tree showed that ABCB1, ABCB4, and ABCB5 were closely related, implying that they may have shared a common ancestor during chordate evolution [24]. Until now, no teleost ABCB1 gene has been detected, suggesting that ABCB1 is absent in teleost fishes. Two copies of ABCB11 (ABCB11a and ABCB11b) were detected in common carp, as well as in zebrafish and medaka. The teleost ABCB11b sequences formed a group with the ABCB11 sequences of chicken and frog, and then clustered with the teleost ABCB11a sequences. The topology suggests that ABCB11a and ABCB11b may be derived from the second round (2R) of WGD instead of from the teleost-specific 3R WGD. The chicken and frog genomes lost the orthologous copies of ABCB11a and have retained only single copy of ABCB11b.

#### ABCC subfamily

**Fig 4:**
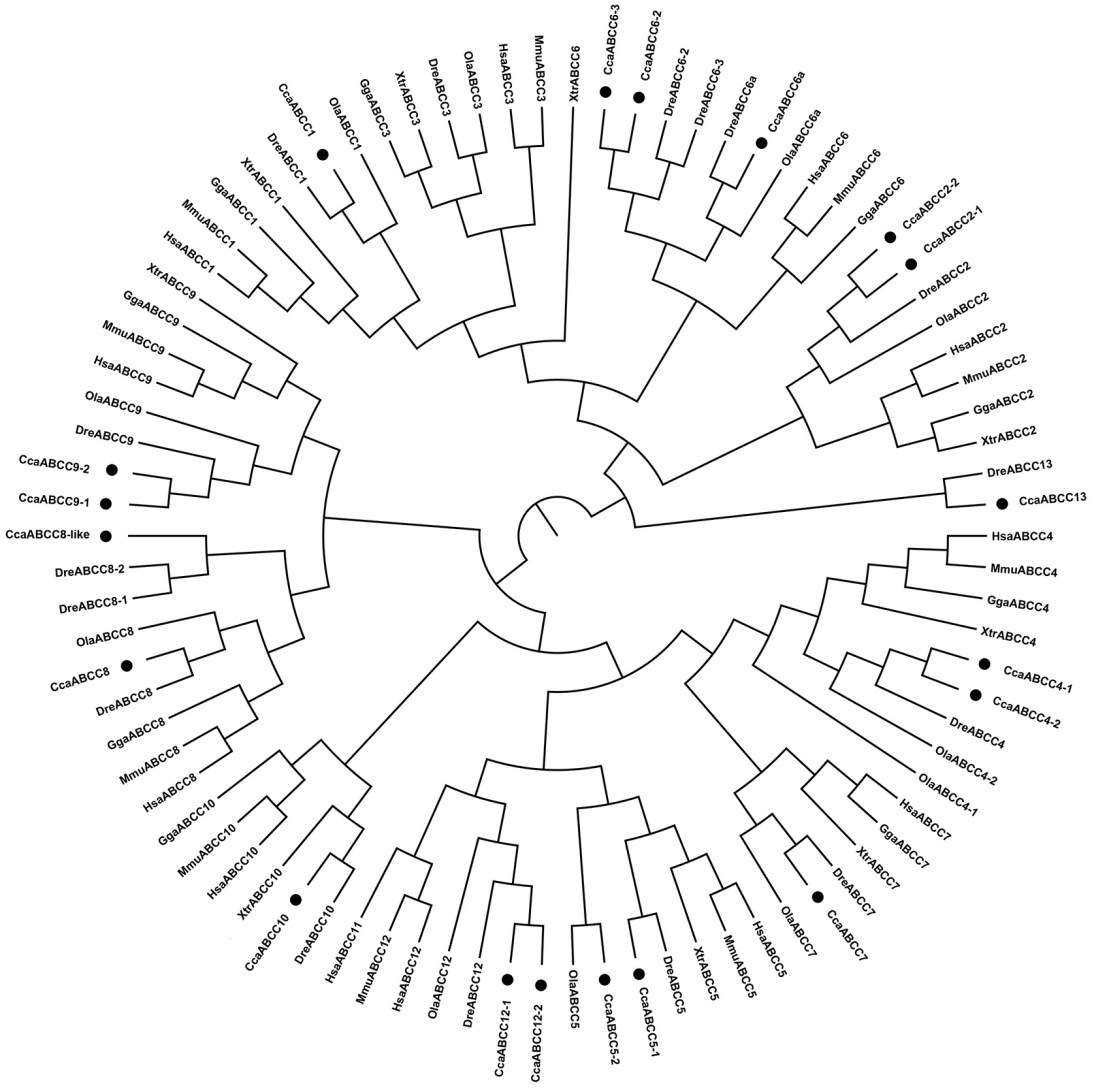
Phylogenetic tree of ABCC subfamily transporters. Neighbor-joining-based phylogenetic tree of ABCC proteins sequences. Human (Hsa), mouse (Mmu), chicken (Gga), frog (Xtr), medaka (Ola), zebrafish (Dre), and common carp (Cca). The black dots indicate common carp genes.

We detected 19 ABCC genes in the common carp genome, which is more that the sixteen in zebrafish and eleven copies in medaka. The common carp ABCC genes were annotated as ABCC1, ABCC2-1, ABCC2-2, ABCC4-1, ABCC4-2, ABCC5-1, ABCC5-2, ABCC6a, ABCC6-2, ABCC6-3, ABCC7, ABCC8, ABCC8-like, ABCC9-1, ABCC9-2, ABCC10, ABCC12-1, ABCC12-2, and ABCC13 (Table 1). Full-length coding sequences as well as partial sequences were obtained for the 19 ABCC transporters (Table 1). The phylogenetic analysis showed that all the common carp ABCC transporters clustered with the orthologs from the other species (Figs 1 and 4). Two copies of ABCC2 (ABCC2-1 and ABCC2-2), ABCC4 (ABCC4-1 and ABCC4-2), ABCC5 (ABCC5-1 and ABCC5-2), ABCC9 (ABCC9-1 and ABCC9-2), and ABCC12 (ABCC12-1 and ABCC12-2) were detected in common carp, probably derived from either the teleost-specific 3R WGD or the latest 4R WGD. We identified three copies of ABCC6 in both the common carp and zebrafish genomes, which were likely derived from the 2R WGD, as well as from segmental duplication. Additional comparative genomic studies will be necessary to unveil the potential mechanisms. We also identified two copies of ABCC8 (ABCC8 and ABCC8-like) in common carp compared with three ABCC8 copies in zebrafish. The DreABCC8-1 and DreABCC8-2 sequences, which were closely related to the common carp ABCC8-like sequence, shared the highest similarity, suggesting they are newly duplicated genes that arose after common carp and zebrafish divergence. In addition, we identified a ABCC13 gene in the common carp genome; until now, ABCC13 was considered to be zebrafish specific [25]. This result suggests that ABCC13 could be a cyprinid-specific rather than a zebrafish-specific gene. The ABCC subfamily includes multidrug resistance-associated proteins that transport diverse substrates including drugs, endogenous compounds, and xenobiotics. In addition to these multidrug resistance proteins, the ABCC subfamily includes the chloride channel ABCC7 (also known as CFTR) and the sulfonylurea receptors ABCC8 and ABCC9 (also known as SUR1 and SUR2 respectively) [26]. The gene expansion of the ABCC subfamily in common carp may facilitate its ability to survive in diverse aquatic environments.

#### ABCD subfamily

**Fig 5:**
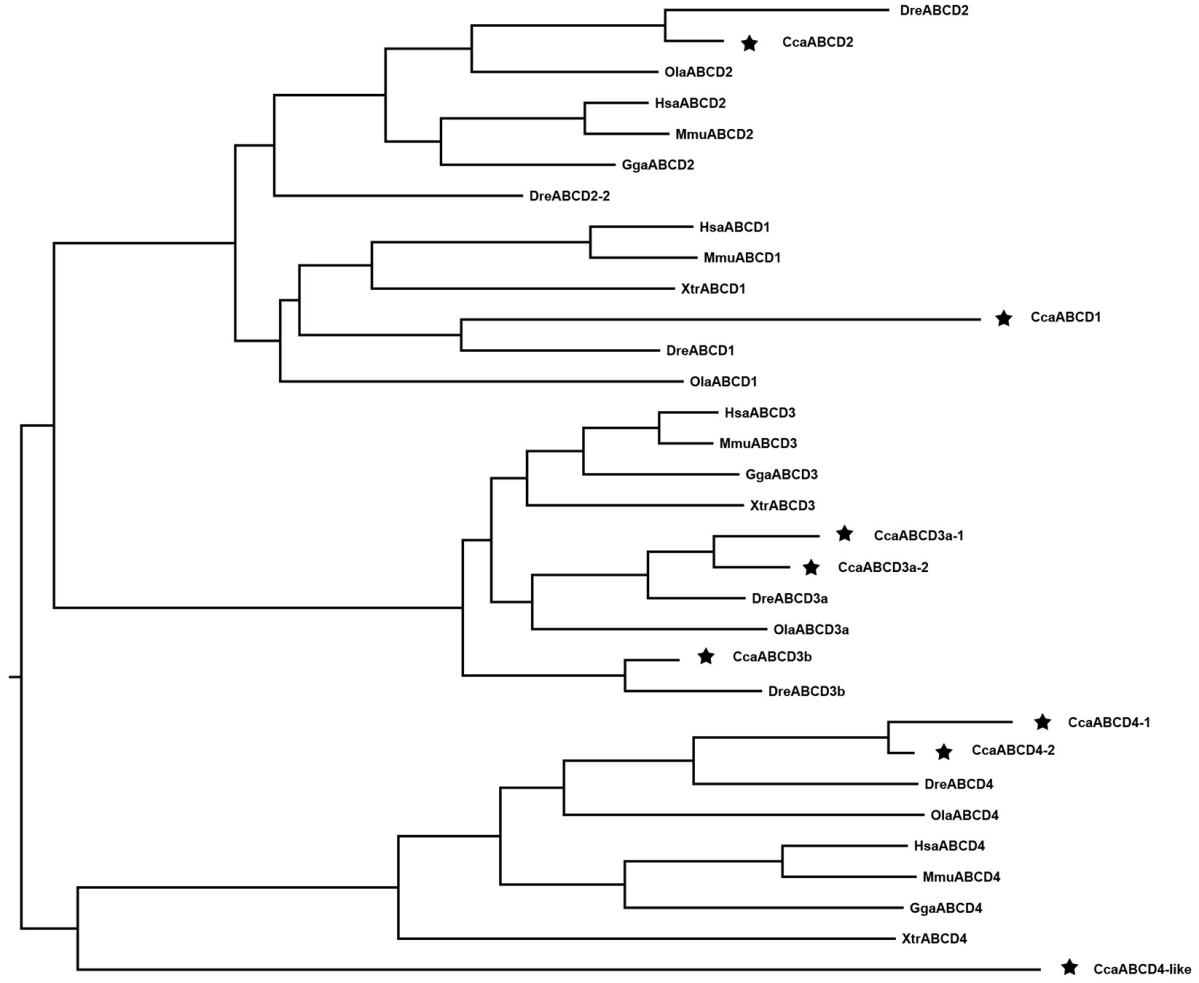
Phylogenetic tree of ABCD subfamily transporters. Neighbor-joining-based phylogenetic tree of ABCD proteins sequences. Human (Hsa), mouse (Mmu), chicken (Gga), frog (Xtr), medaka (Ola), zebrafish (Dre), and common carp (Cca). The black stars indicate common carp genes.

The eight ABCD genes identified in the common carp genome were annotated as ABCD1, ABCD2, ABCD3a-1, ABCD3a-2, ABCD3b, ABCD4-1, ABCD4-2, and ABCD4-like (Table 1), which is considerably more than the four and five ABCD genes that have been identified in the medaka and zebrafish genomes respectively. Full-length coding sequences as well as partial sequences were obtained for the eight ABCD transporters (Table 1). The phylogenic analysis suggested that the ABCD3b sequences from zebrafish and common carp may be more ancient than the ABCD3a sequences, which may be derived from the 2R WGD in vertebrates (Figs 1 and 5). Although the other studied vertebrates, from teleost fishes to mammals, retained only ABCD3a in their genomes, the cyprinids retained the more ancient ABCD3b. The two ABCD3a copies (ABCD3a-1 and ABCD3a-2) are probably young homologs derived from the latest 4R WGD.

#### ABCE/F subfamily

**Fig 6:**
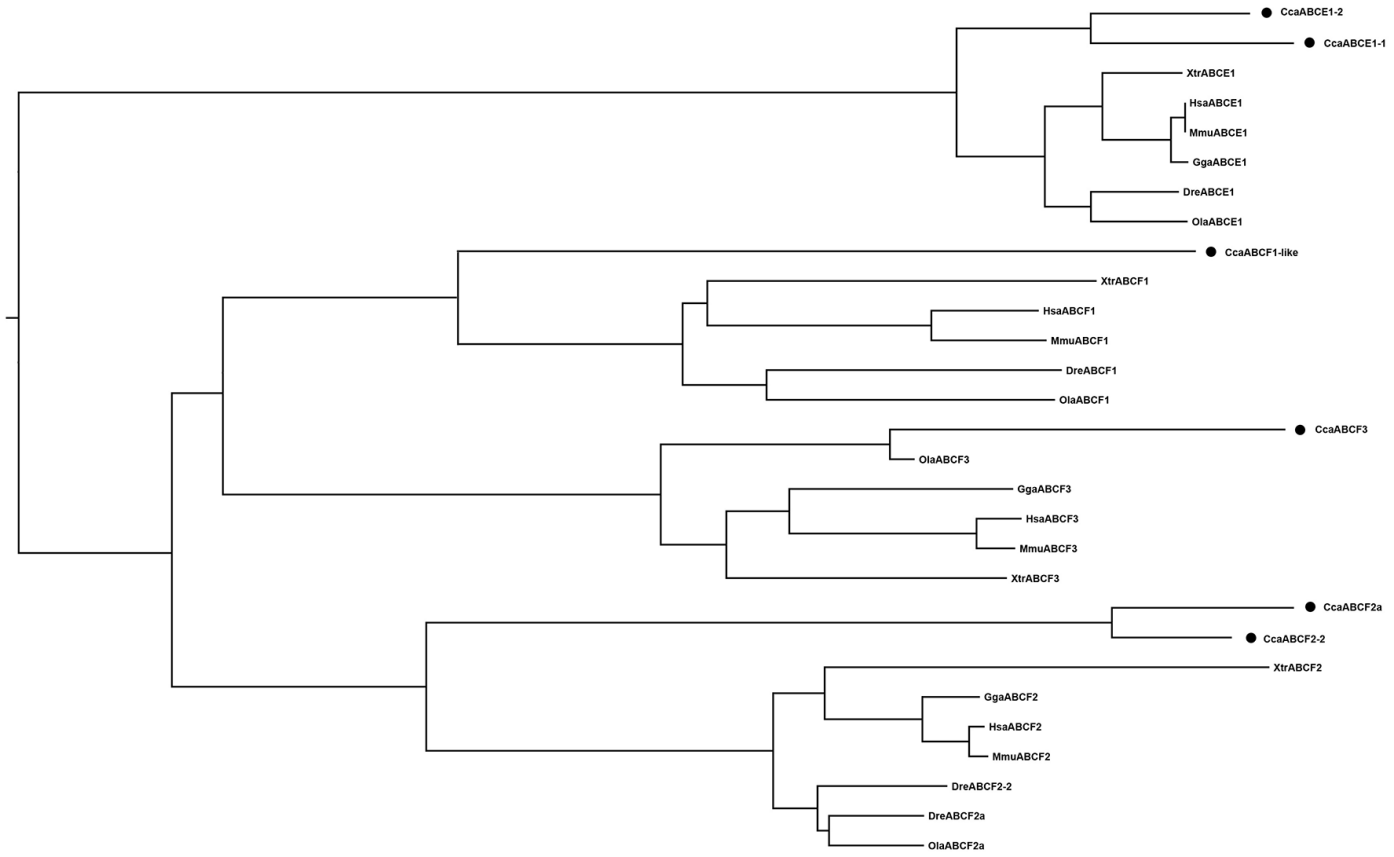
Phylogenetic tree of ABCE/F subfamily transporters. Neighbor-joining-based phylogenetic tree of ABCE/F proteins sequences. Human (Hsa), mouse (Mmu), chicken (Gga), frog (Xtr), medaka (Ola), zebrafish (Dre), and common carp (Cca). The black dots indicate common carp genes.

All the ABCE and ABCF proteins contained two NBDs but no TMDs, making them non-functional as transporters [27]. Four members of the ABCE/F subfamily have been identified in vertebrates. We identified six ABCE/F genes in the common carp genome, with gene duplications only in ABCE1 and ABCF2 (Table 1, Figs 1 and 6).

#### ABCG/H subfamily

**Fig 7:**
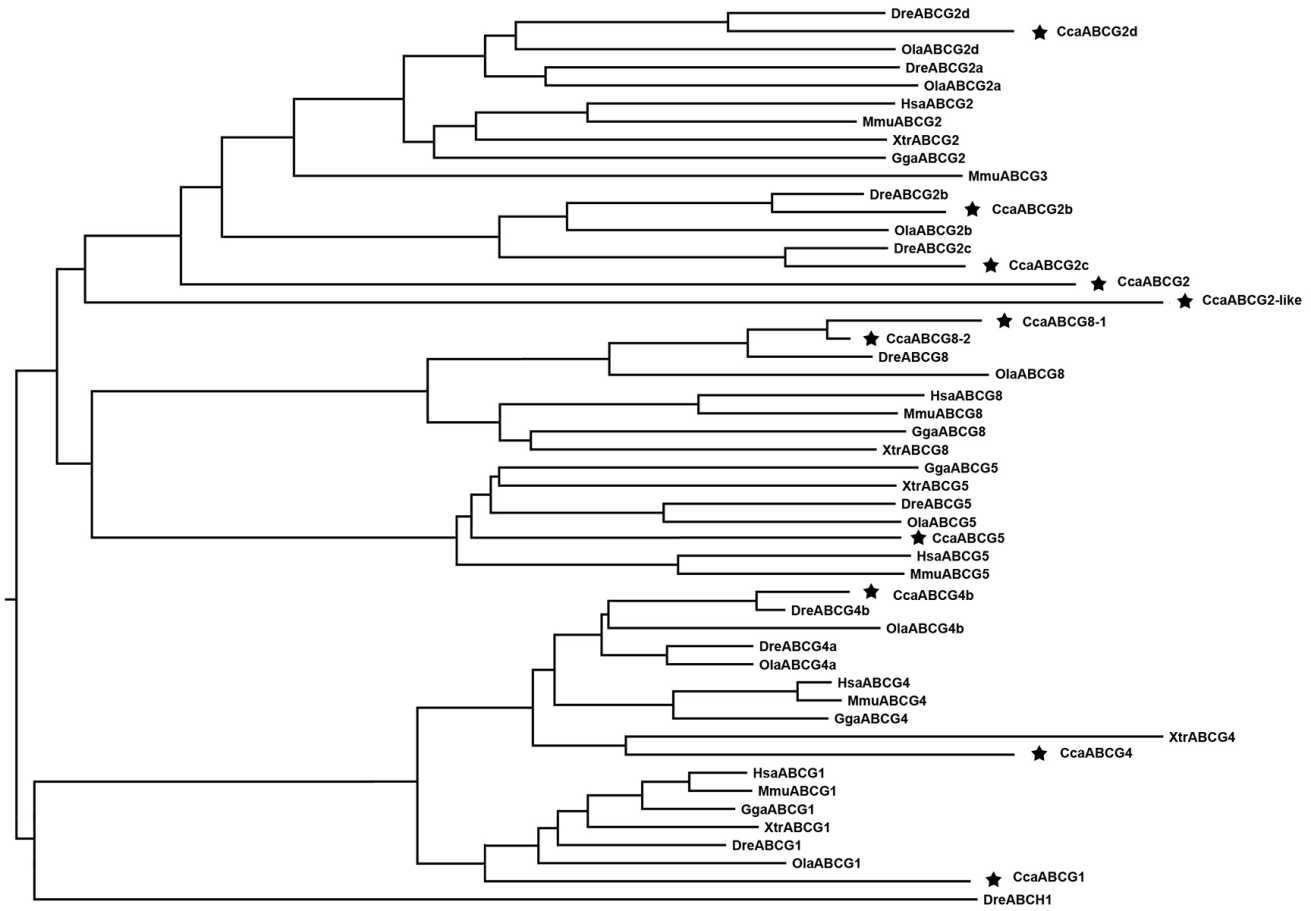
Phylogenetic tree of ABCG/H subfamily transporters. Neighbor-joining-based phylogenetic tree of ABCG/H proteins sequences. Human (Hsa), mouse (Mmu), chicken (Gga), frog (Xtr), medaka (Ola), zebrafish (Dre), and common carp (Cca). The black stars indicate common carp genes.

Five members of the ABCG subfamily have been identified in vertebrates. We identified 11 ABCG genes in the common carp genome, which were annotated as ABCG1, ABCG2, ABCG2b, ABCG2c, ABCG2d, ABCG4, ABCG4b, ABCG5, ABCG8-1, ABCG8-2, and ABCG2-like (Table 1). Full-length coding sequences were obtained for the 11 ABCG/H transporters (Table 1). The phylogenic analysis showed that extensive ABCG2 gene expansion had taken place in the three teleost fishes (Figs 1 and 7). Five ABCG2 copies (ABCG2, ABCG2b, ABCG2c, ABCG2d, ABCG2-like) were found in common carp compared with four ABCG2 copies in zebrafish and three in medaka. The ABCG2 transporter has been reported to be a high-capacity urate exporter [28]. The ABCG2 gene expansion may be an important mechanism for living in aquatic environments. In addition, two copies of ABCG4 (ABCG4 and ABCG4b) and ABCG8 (ABCG8-1, ABCG8-2) were identified in the common carp genome. Furthermore, we identified an orphan copy of ABCH (ABCH1) in the zebrafish genome. The presence of the ABCH subfamily in teleost fish is still controversial. Until now, the ABCH gene has been annotated only in zebrafish and a putative form was identified in green spotted pufferfish, which was confirmed in a review of ABC drug transporters [29]. Our analyses did not detect any ABCH homologs in any of the surveyed vertebrate genomes, which is consistent with a previous report [14].

### Gene duplications and losses of ABC transporter genes in common carp

WGD is one of the main driving forces in the evolution of many vertebrates because it produces an enormous number of new genes with the potential for new functions. The high diversification of teleost fish is supposed to correlate with the teleost-specific 3R WGD that took place in the common ancestor of all extant teleosts. As a result, teleost fish have two paralogous copies for many genes, while only one ortholog is present in tetrapods [30]. Common carp retained 100 chromosomes. It has been suggested that the 4R WGD may have occurred around 8.2 million years ago [31]. Analysis of microsatellite loci [32] and comparative analysis of the common carp and zebrafish linkage maps [33] have provided critical evidence in support of the 4R WGD in common carp.

**Fig 8:**
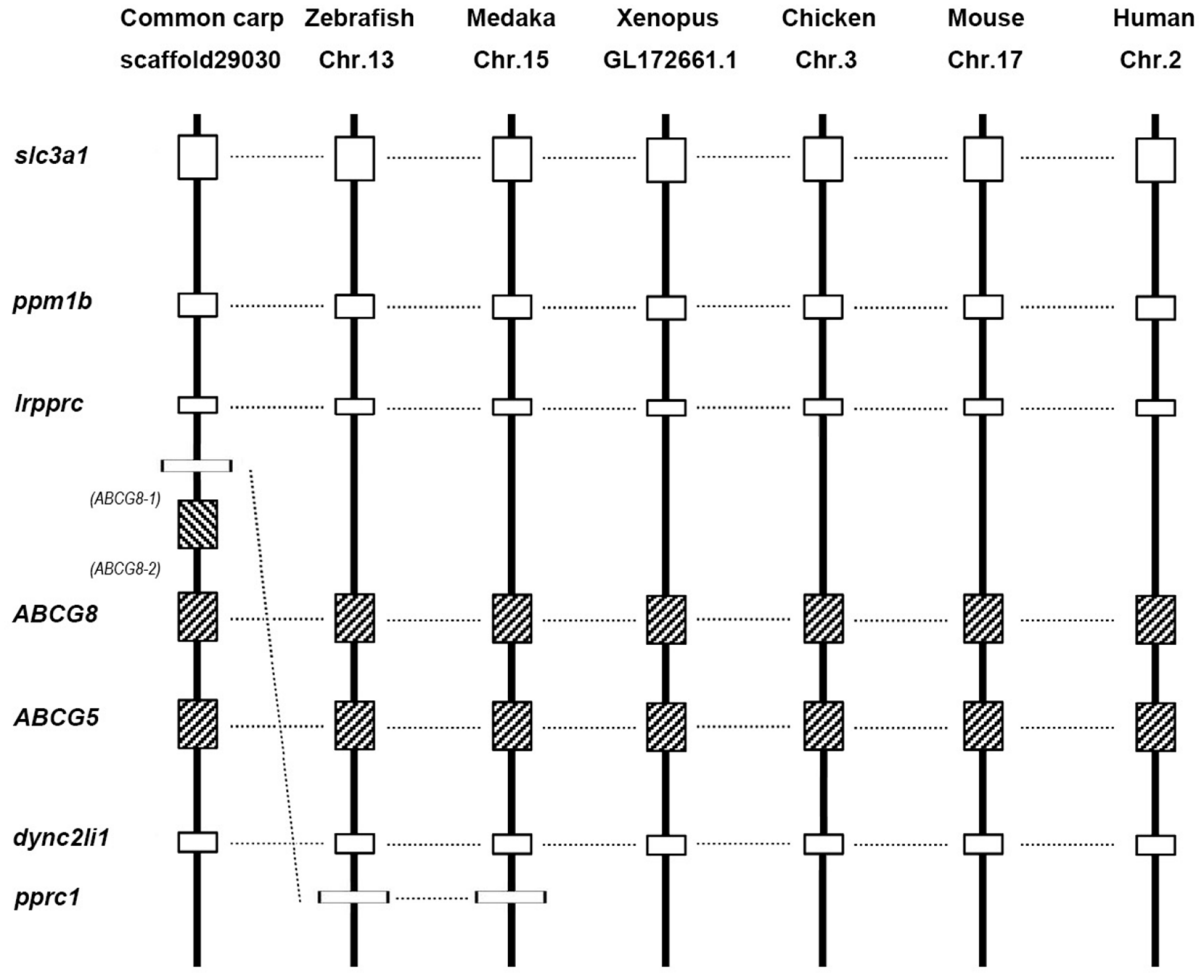
Conserved synteny blocks harboring the ABCG8 gene in vertebrate genomes. Horizontal lines indicate orthologous relationships. Gene names: slc3a1 (solute carrier gene family 3, member 1); ppm1b (protein phosphatase, Mg2^+^/Mn2^+^ dependent, 1B); lrpprc (leucine-rich pentatricopeptide repeat containing); ABCG8 (ATP-binding cassette, sub-family G, member 8); ABCG5 (ATP-binding cassette, sub-family G, member 5); dync2li1 (dynein, cytoplasmic 2, light intermediate chain 1); pprc1 (peroxisome proliferator-activated receptor gamma coactivator-related protein 1).

In this study, we examined the copy numbers of ABC genes in several vertebrate genomes and inferred that duplicate copies of ABCA1, ABCA4, ABCB11, ABCC6, ABCC8, ABCD3, ABCF2, ABCG2, and ABCG4 derived from the 3R WGD were retained in the diploid teleosts (medaka or/and zebrafish) (Table 2). Lineage-specific duplication events generally result from segmental duplication, especially tandem duplication. Tandem duplication usually generates tandem genes or gene clusters in one chromosome or scaffold. Syntenic analysis of ABCG8 paralogs across all the vertebrates revealed that the genes were located in the same scaffold of the common carp genome, implying they were derived from tandem duplication rather than WGD (Fig 8). Moreover, we also found that ABCG5 was closely related to ABCG8 in all the surveyed species.

**Table 2:** Comparison of the ABC transporter subfamilies in seven vertebrate species including common carp.

Gene name	Chicken	Xenopus	Human	Mouse	Medaka	Zebrafish	Carp
**ABCA1**	1	1	1	1	2	2	4
**ABCA2**	1	1	1	1	1	1	1
**ABCA3**	1	1	1	1	1	1	1
**ABCA4**	1	1	1	1	1	2	2
**ABCA5**	1	1	1	1	1	1	2
**ABCA6**	0	0	1	1	0	0	0
**ABCA7**	1	0	1	1	1	1	0
**ABCA8**	1	1	1	2	0	0	0
**ABCA9**	0	1	1	1	0	0	0
**ABCA10**	0	1	1	0	0	0	0
**ABCA12**	1	1	1	1	1	1	1
**ABCA13**	1	0	1	1	0	0	0
**ABCA14**	0	0	0	1	0	0	0
**ABCA15**	0	0	0	1	0	0	0
**ABCA16**	0	0	0	1	0	0	0
**ABCA17**	0	0	0	1	0	0	0
**ABCB1**	2	1	1	2	0	0	0
**ABCB2**	0	0	0	0	0	0	0
**ABCB3**	0	0	0	0	0	1	0
**ABCB4**	0	0	1	1	1	1	1
**ABCB5**	0	1	1	1	0	1	2
**ABCB6**	1	1	1	1	1	2	0
**ABCB7**	1	1	1	1	1	1	0
**ABCB8**	0	1	1	1	1	1	0
**ABCB9**	1	1	1	1	1	1	1
**ABCB10**	1	1	1	1	1	1	0
**ABCB11**	1	1	1	1	2	2	2
**Tap1**	1	1	1	1	1	1	0
**Tap2**	1	1	1	1	0	0	0
**ABCC1**	1	1	1	1	1	1	1
**ABCC2**	1	1	1	1	1	1	2
**ABCC3**	1	1	1	1	1	1	0
**ABCC4**	1	1	1	1	2	1	2
**ABCC5**	0	1	1	1	1	1	2
**ABCC6**	1	1	1	1	1	3	3
**ABCC7**	1	1	1	0	1	1	1
**ABCC8**	1	0	1	1	1	3	2
**ABCC9**	1	1	1	1	1	1	2
**ABCC10**	1	1	1	1	0	1	1
**ABCC11**	0	0	1	0	0	0	0
**ABCC12**	0	0	1	1	1	1	2
**ABCC13**	0	0	0	0	0	1	1
**ABCD1**	0	1	1	1	1	1	1
**ABCD2**	1	0	1	1	1	2	1
**ABCD3**	1	1	1	1	1	2	3
**ABCD4**	1	1	1	1	1	1	3
**ABCE1**	1	1	1	1	1	1	2
**ABCF1**	0	1	1	1	1	1	1
**ABCF2**	1	1	1	1	1	2	2
**ABCF3**	1	1	1	1	1	0	1
**ABCG1**	1	1	1	1	1	1	1
**ABCG2**	1	1	1	1	3	4	5
**ABCG3**	0	0	0	1	0	0	0
**ABCG4**	1	1	1	1	2	2	2
**ABCG5**	1	1	1	1	1	1	1
**ABCG8**	1	1	1	1	1	1	2
**ABCH1**	0	0	0	0	0	1	0
**Total**	38	40	48	52	44	57	61

**Fig 9:**
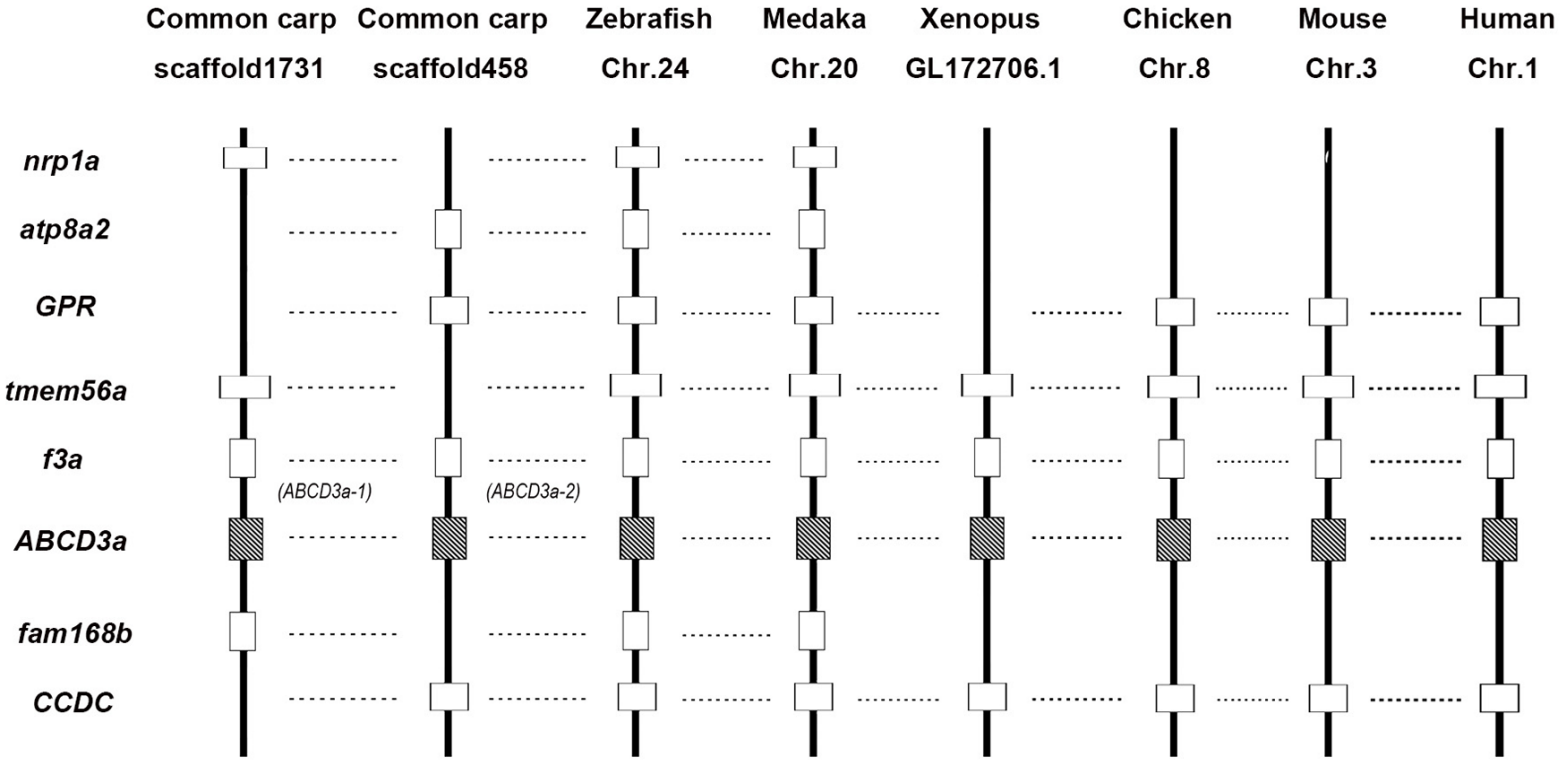
Conserved synteny blocks harboring the ABCD3a gene in vertebrate genomes. Horizontal lines indicate orthologous relationships. Gene names: nrp1a (neuropilin 1a); atp8a2 (ATPase, aminophospholipid transporter, class I, type 8A, member 2); GPR (G protein-coupled receptor); tmem56a (transmembrane protein 56a); f3a (coagulation factor IIIa); ABCD3a (ATP-binding cassette, sub-family D, member 3a); fam168b (family with sequence similarity 168, member B); CCDC (coiled-coil domain-containing protein).

We found that the common carp ABCA1, ABCA5, ABCB5, ABCC2, ABCC4, ABCC5, ABCC9, ABCC12, ABCD4, ABCE1, and ABCG8 genes had undergone significant numbers of duplications compared with their orthologs in the closely related zebrafish genome, which suggested that they these genes may be derived from the 4R WGD (Table 2). To test this, we constructed syntenic blocks of ABCD3a and found that the two copies of ABCD3a were located on two different scaffolds, which clearly demonstrated their WGD origin in common carp (Fig 9).

Lineage-specific gene losses were also observed in common carp; indeed, ABCA6, ABCA8/9/10, ABCA13/14/15/16/17, ABCB1/2, TAP2, ABCC11, and ABCG3 genes were not found in any of the fish genome (Table 2). These non-fish ABC genes must have appeared as a result of duplications that occurred after the split of tetrapods from teleost fishes. Among them, ABCA14/15/16/17 and ABCG3 were found only in mouse, ABCC11 was found only in human, and ABCA6 was found only in the mammalian species, while ABCA8 and TAP2 were found in all the surveyed species except the teleosts [34]. Interestingly, ABCC13 was found in the zebrafish and common carp genomes when previously it was believed to be present only in the dog and macaque genomes [34].

### Expression of ABC transporter genes in common carp

The unexpected expansion of the ABC transporter gene family that we detected in common carp raises the question of how many of these genes are actually expressed. The expression patterns of these genes together with information about their orthologous in model species should allow functional inferences to be made. We conducted RT-PCRs using gene-specific primers to examine the expression patterns of each the ABC transporter genes in six common carp tissues. In general, most of the ABC transporter genes were widely expressed, but their expression levels were different in different tissues.

#### ABCA subfamily

**Fig 10:**
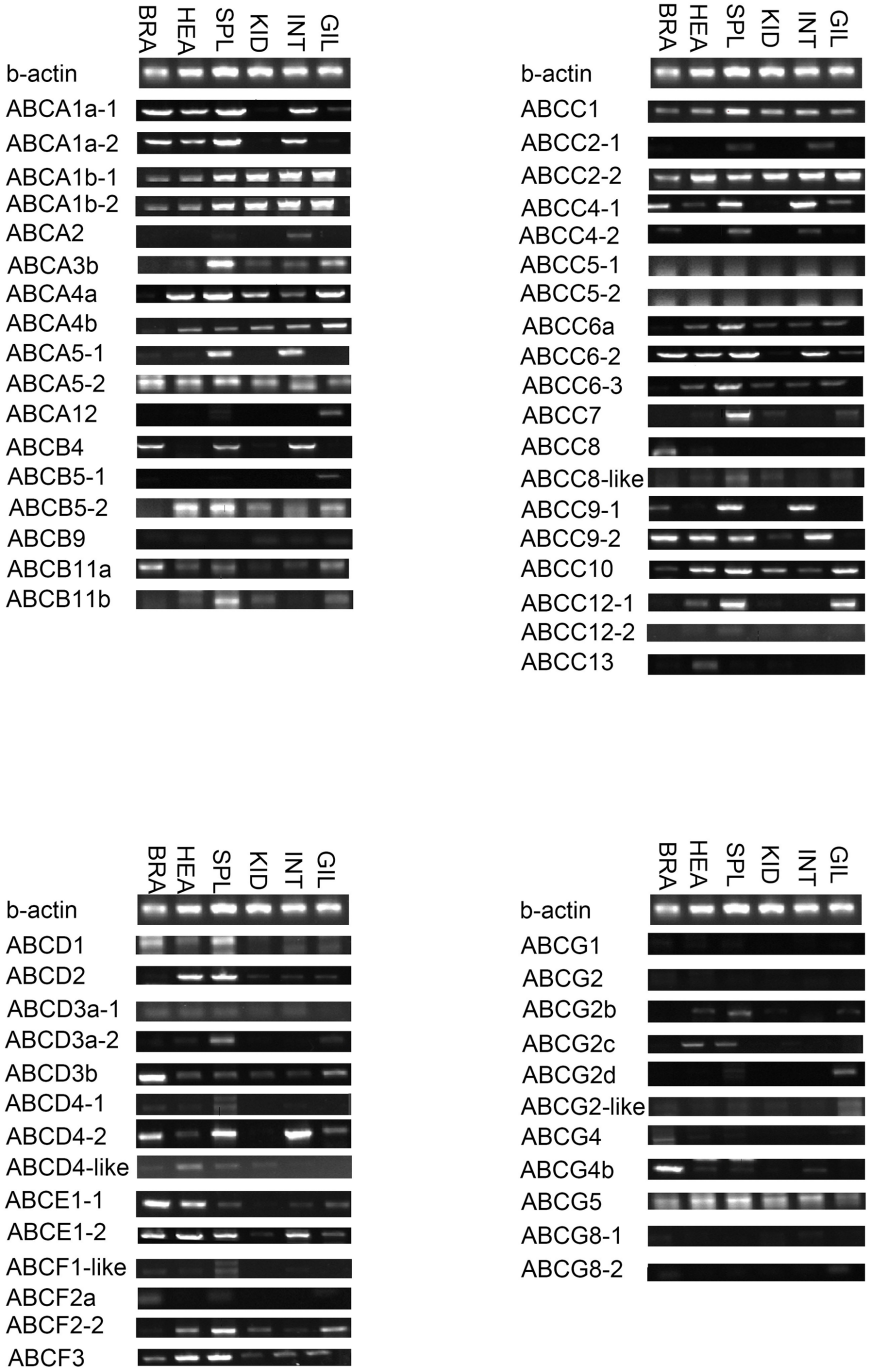
Expression levels of common carp ABC transporter genes in six tissues by RT-PCR. β-actin was used as an internal control. Gene names are indicated on the left of the panels. The six tissues are: BRA (brain), HEA (heart), SPL (spleen), KID (kidney), INT (intestine), and GIL (gill).

Nine the ABCA transporter genes were ubiquitously expressed in the six common carp tissues tested, while ABCA2 and ABCA12 were expressed in only two tissues (Fig 10). The four ABCA1 genes were expressed in all the tissues; in human, ABCA1 has been reported to play a significant role in cholesterol efflux [35]. ABCA3 was highly expressed in gill; in human, its expression was found to by developmentally regulated, peaking prior to birth under the influence of steroids and transcription factors [36]. The two copies of ABCA5 had different expression in each of the tissues, especially in spleen and intestine, and ABCA5 has been reported to regulate amyloid-beta peptide production in human and mouse brain [37]. In addition, a mutation in the ABCA12 gene was shown to regulate Harlequin ichthyosis [38]. ABCA4 was highly expressed in all of the common carp tissues except brain. In human, ABCA4 acts as a transporter of *N*-retinylidene-phosphatidylethanolamine (NrPE), and a lack of ABCA4 was reported to lead to the formation of lipid deposits in the macular region of the retina [39].

#### ABCB subfamily

The six common carp ABCB transporter genes were found to be widely expressed, although ABCB9 expression was very low in all six tissues tested (Fig 10). The two ABCB5 copies had different expressions in each tissue, especially in gill. In a cell-based study, ABCB5 was shown to identify immunoregulatory dermal cells and thus suppressed T cell proliferation [40]. ABCB4 was relatively highly expressed in brain, spleen, and intestine. The two ABCB11 copies were expressed at average levels in the six tissues tested. A novel ABCB11 mutation was reported to be associated with benign recurrent intrahepatic cholestasis in human [41]. Moreover, some members of the plant ABCB subfamily have been found to display very high substrate specificity compared with mammalian ABCBs, which are often associated with multidrug resistance [1].

#### ABCC subfamily

The 19 common carp ABCC transporter genes were widely expressed, although the expression of ABCC2-1 and ABCC12-2 was very low (Fig 10). ABCC1 was highly expressed in all six tissues. In human, overexpression of ABCC1 was found to lead to multidrug resistance, especially during cancer and leukemia chemotherapy treatments [42]. The two ABCC2 copies were expressed differently in each of the tissues, especially in spleen and intestine. In a human cell-based study, ABCC2 was found to be responsible for the transport of conjugated bilirubin through the plasma membrane [43]. ABCC4 and ABCC5 had low expression levels while ABCC10 had high expression levels in the six tissues tested. The three ABCC6 copies were highly expressed in all six tissues. ABCC8 and ABCC9 are sulfonylurea receptors, which, in human, were found to be the molecular targets of the sulfonylurea class of anti-diabetic drugs [44]. In addition, highly prevalent point mutations in the chloride ion channel ABCC7 gene have been shown to cause cystic fibrosis [9]. We found that ABCC13 was highly expressed in common carp heart. In a previous study, an unusual truncated ABC transporter was reported to be highly expressed in fetal human liver [45].

#### ABCD subfamily

The eight common carp ABCD transporter genes were expressed in the six tissues tested, although their expression levels were relatively low (Fig 10). Members of the ABCD subfamily are thought to localize in the peroxisomal membrane, endoplasmic reticulum, or lysosomes [46]. In mammalian cells, peroxisomes are involved in a number of important metabolic pathways, including the α- and β-oxidation of fatty acids and the biosynthesis of phospholipids and bile acids. Substrates for β-oxidation enter peroxisomes via ABCD transporters and are activated by specific acyl-CoA synthetases for further metabolism [47]. Mammalian ABCDs are also thought to be responsible for adrenoleukodystrophy, which is an X chromosome-linked disease [10].

#### ABCE/F subfamily

The six common carp ABCE/F transporter genes were widely expressed in all six tissues, although the expressions of ABCF1-like and ABCF2a were slightly different, as they just expressed in specific tissues (Fig 10). Because the ABCE and ABCF proteins contain a pair of NBDs but no TMDs, they are probably non-functional as transporters.

#### ABCG/H subfamily

Ten of the 11 common carp ABCG transporter genes had very weak expression in all six tissues; ABCG5, which was highly expressed in all tissues, was the exception (Fig 10). Similar to the multidrug resistance proteins, ABCB1 and ABCC1, the multidrug resistance ABCG2 transporters have been shown to transport diverse therapeutic drugs [48]. A recent study revealed that ABCG2 showed enhanced expression in side population cells that exist around cancer stem cells [49]. The five ABCG2 copies had different expressions in each of the common carp tissues tested, especially in heart and spleen, which implies a tissue-specific pattern of gene expression. The two ABCG4 copies were expressed highly only in brain. In plants, ABCG5 and ABCG8 transport sterols including cholesterol [11].

## Materials and Methods

### Ethic Statement

This study was approved by the Animal Care and Use committee of Centre for Applied Aquatic Genomics at Chinese Academy of Fishery Sciences. The methods were carried out in accordance with approved guidelines. Adult common carp were collected from the Breeding Station of Henan Academy of Fishery Research, Zhengzhou, Henan province, China. Euthanasia is performed by immersion fish in MS-222 solution. Tissue samples of brain, heart, gill, intestine, kidney and spleen were collected from 10 individuals and immediately placed in 2 ml RNAlater (Qiagen, Hilden, Germeny) and kept at -20°C until RNA extraction.

### Identification of ABC transporter genes and homologs

All available ABC transporter gene sequences of zebrafish (*Danio rerio*) were downloaded from the Ensembl Zebrafish database (http://asia.ensembl.org/Danio_rerio/info/index). BLAST searches (with E-value cutoff of 1e−5) were conducted against the whole genome sequences, annotated genes, and transcriptome contigs of common carp to obtain candidate ABC genes. Reciprocal BLAST searches were conducted to verify the veracity of the candidate genes. Coding sequences were confirmed by BLAST searches against the NCBI non-redundant protein sequence database. Full-length translated amino acid sequences as well as the partial sequences coding for the conserved domains were used in the phylogenetic analyses. Full-length ABC protein sequences from other vertebrate species were retrieved from the Ensembl genome database (Release 82) for the phylogenetic analyses.

### Phylogenetic analysis of ABC transporter genes

Phylogenetic analysis can be used to support gene annotations, especially for non-model species. Reference ABC transporter genes from representative vertebrate model species were used for phylogenetic analysis, including *Homo sapiens* (human), *Mus musculus* (mouse), *Gallus gallus* (chicken), *Xenopus tropicalis* (frog), *Oryzias latipes* (medaka), and *Danio rerio* (zebrafish) for the phylogenetic analyses. The amino acid sequences were aligned using ClustalW (http://www.clustal.org/clustal2) with the default parameters. Several neighbor-joining phylogenetic trees were constructed using Clustal Omega (http://www.ebi.ac.uk/Tools/msa/clustalo/) with default settings of Pairwise Alignment and Multiple Sequence Alignment. Each common carp ABC protein was assigned to a family based on the phylogenetic analysis with the human and zebrafish ABC protein sequences. Separate phylogenetic trees were constructed for each ABC subfamily using the same methodology with other representative vertebrate species.

### Nomenclature of the ABC transporter genes in common carp

The common carp ABC orthologous genes were named based on the topologies of the phylogenetic trees, as well as the most closely related zebrafish genes. Then, the closely related zebrafish ABC genes were assigned to each common carp ABC ortholog and the ABC genes were named after their most closely related zebrafish gene (for instance, ABCB11a, ABCB11b). Besides, we annotated ABCs that had more than two copies in the common carp genome with the postscript “-1” or “-2” following the name of zebrafish orthologs to reflect the fourth round (4R) of WGD [50] (For example, ABCA1a-1 and ABCA1a-2). In addition, some common carp ABCs were assigned the postscript “-like”.

### Syntenic analysis of ABC transporter genes

Syntenic analysis was performed on selected ABC genes in human, mouse, chicken, frog, medaka, zebrafish, and common carp chromosomes/scaffolds by identifying the positions of neighboring ABC genes. Syntenic maps were drawn based on the organization of the genes on the chromosomes of the model species from the Ensembl databases, and the gene organization of common carp was according to the draft common carp genome assembly.

### Expression of ABC transporter genes

Total RNA was extracted from six adult common carp tissues (brain, heart, spleen, kidney, intestine, and gill) using Trizol reagent (Life Technologies, NY, USA), and cDNA was synthesized by RT-PCR using a SuperScript III Synthesis System (Life Technologies). The ß-actin gene was used as an internal positive control, with forward primer (5′-TGCAAAGCCGGATTCGCTGG-3′) and reverse primer (5′-AGTTGGTGACAATACCGTGC-3′). The PCR amplification comprised an initial denaturation step for 5 min at 95°C followed by 30 cycles of denaturation (30 sec at 94°C), annealing (30 sec at 60°C), and extension (20 sec at 72°C), and a final elongation step of 5 min at 72°C. The primers were listed in S2 Table. The PCR products were separated by gel electrophoresis (1% agarose gel at 150 V) in the presence of ethidium bromide and visualized under ultraviolet light.

## Conclusions

In this study, we identified a total of 61ABC transporter genes in the tetraploid common carp genome. Phylogenetic analysis and comparative genomic study provided a comprehensive understanding of the ABC gene family and their distribution in the genome. Both 3R and 4R WGDs are inferred during the analysis. While the great majority of ABC transporters are well conserved through evolution, identification and phylogenetic analysis of the ABC transporters in common carp produced some interesting results. 1) Ten ABC transporter genes were duplicated in the teleost genomes, namely ABCA1, ABCA4, ABCB11, ABCC6, ABCC8, ABCD3, ABCF2, ABCG2, ABCG4, and ABCG8, indicating a teleost-specific 3R WGD occurred in these fishes. 2) Twelve ABC transporter genes were duplicated in the common carp genome compared with the zebrafish genome, namely ABCA1, ABCA5, ABCB5, ABCC2/4/5/9/12, ABCD3/4, ABCE1, and ABCG8, which implies a 4R WGD event occurred in common carp. 3) Fourteen ABC transporters have not yet been identified in any of the fish genomes studied, namely ABCA6/8/9/10/13/14/15/16/17, ABCB1, two copies of TAP, ABCC11, and ABCG3, suggesting lineage-specific gene losses occurred from the teleost genomes. 4) ABCC13 was detected in the common carp and zebrafish genomes, although it has not yet been detected in other teleost fishes.

We also performed RT-PCRs to determine the expression patterns of the common carp ABC transporter genes. Most of the ABC genes were ubiquitously expressed in six tissues from common carp. The exceptions were the ABCG genes, most of which were weakly expressed. Different gene copies were differently expressed in some tissues, indicating tissue-specific gene functions may be present to some extent. However, the detailed functions of each of the genes need further study. This study provides essential genomic resources for future biochemical, toxicological, physiological and evolutionary studies in common carp.

## Supporting Information

S1 Table All common carp ABC transporter family sequences. (DOCX)

S2 Table All common carp ABC gene primer sequences. (DOCX)
