# Supplementary material for: Genome-Wide Identification, Characterization and Phylogenetic Analysis of ATP-Binding Cassette (ABC) Transporter Genes in Common Carp (Cyprinus carpio)
Source: PLoS One. 2016 Apr 8;11(4):e0153246. doi: 10.1371/journal.pone.0153246 (PMC4825979; doi:10.1371/journal.pone.0153246)
Supplement: S1 Table — (DOCX) [file pone.0153246.s001.docx]

**S1 Table. All common carp ABC transporter family sequence.**

| **Gene name** | **Protein Sequence** |
| --- | --- |
| CcpABCA1a-1 | MSRSMPLFMTLAWMYSVAIIIKGVVYEKEARLKETMRIMGLDNGILWLSWFISSLIPLLISAALLVLILKMGNLLPYSDPGVVYLFLASFAVVTIMQCFLISTLFSRANLAAACGGIIYFTLYLPYVLCVAWQDYVGFGAKVVVSLLSPVAFGFGCEYFALFEEQGEDSVSESSSDAGLGSDHESETATIDVSLISNVIFKHVPTARLVEDLGHEITYVLPYESAKDGAFVELFHEIDDRLTDLGISSYGISDTTLEEIFLKVAEDNGVDAEMSDGIIPARRNRRHAFGDHQSCLKPFTEDDFDFNDSEGDPDGIIPARRNRRHAFGDHQSCLKPFTEDDFDFNDSEGDPESRETDWLGGADGKGSYQVKGWSLKRQQFVALLWKRFLYARRSRKGFFAQIVLPAVFVCIALVFSLIVPPFGKYPSLALEPSMYEEQFTFISNDAPEDRHTNKLLEALMNNPAYNEQCKDEQNIVSKSCPIKDGDWMVPEVPESVQDIFLKGNWSIENPSPMCECSCERRKKMLPECPPGAGGLPPPQIKVTVNETLQNLTGKNISDYLVKTYAQIIGKSFLKNKLWVNEFRYGGFSLGARSSQALPPGDEITDAISHIRKRFSLQEGTAPDRFLASLSIFIRGLDTKNNVKIWFNNKGWHSIGGFLNVMNNAVLRANLPPAWISPNSGSKRSTIRSTSPKSSSHRMTTSVDVLVSICVIFAMSFVPASFVVFLIQERVNKAKHMQFISGVQPLLYWLANFVWDMCNYIVPATLVIIIFVCFQQEAYVSSTNLPVLALLLLLYGWSITPLMYPASFFFKIPSTAYVVLTSVNILIGINGSVSTFVLELFGSNEIGGVNDILKNVFLIFPHFCLGRGLIDMVKNQAMADALERFGENRFRSPLAWDMVGKNLFAMAIEGVIFFCITILIQYRFCIKARPVSTKLMPIGEEDEDVARERQRILTGGGHTDILEIKQLTKVADWGIRKLGLMKYVDKAAGSYSGGNMRKLSTAMALIGGPPVVFLDEPTTGMDPKARRALWNCILSIIKEGRSVVLTSH |
| CcpABCA1a-2 | MGNLLPYSDPGVVYLFLASFAVVTIMQCFLISTLFSRANLAAACGGIIYFTLYLPYVLCVAWQDYVGFGAKVVVSLLSPVAFGFGCEYFALFEEQGVGIQWSNLLSSPMQEDSYSLTTSISLMLFDSVLYAVMTWYIEAVFPDISLISNVIFKHVPTARLVEDLGHEITYVLPYESAKDGAFVELFHEIDDRLTDLGISSYGISDTTLEEIFLKVAEDNGVDAEMSDGIIPARRNRRHAFGDHQSCLKPFTEDDFDFNDSEGDPESRETDWLGGADGKGSYQILTCNILTNASFQIVLPAVFVCIALVFSLIVPPFGKYPSLALEPSMYEEQFSFISSKSCPNKDGEWMVPEIPETVQDIFVKGNWSMENPSPLCECSCEGRKRMLPECPPGAGGLPPPQIKVTINETLQNLTGRNISDYLVKTYAQIIGKSLKNKLWVNEFRYGGFSLGARSSQALPPGDEITDAISHIRNRFSLQAGTAADRFLGSLSTFIRGLDTKNNVKIWFNNKGWHSIGAFLNVMNNAVLRATLPPGLDQSKFGIKAFNHPLKPHQRAALTGGTERVNKAKHMQFISGVQPFLYWLANFVWDMCNYIVPATLVIIIFVCFQQEAYVSSTNLPVLALLLLLYGWSITPLMYPASFFFKIPSTAYVVLTSVNILIGINGSVSTFVLELFGSNEIGGINDILKNVFLIFPHFCLGRGLIDMVKNQAMADALERFGENRFRSPLAWDMVGKNLFAMAIEGVIFFCITVLIQYRFCIKARSVSTKLKPIGEEDEDVARERQRILCGGGQTDILELKQLTKVADWGIRKLGLMKYVDKAAGSYSGGNMRKLSTAMALIGGPPVVFLDEPTTGMDPKARRALWNCILSIIKEGRSVVLTSHSMEECEALCTRMAIMVNGRFRCLGSVQHLKNKFGDGYTIILRVAGADPQLEPVMEFIERELPGSTLKEKHRNMLQYQLPSSLTSLARIFSLLSKNKEQLHIEDYSVSQTTLDQVFVNFAKDQSDEDHLKDISINKNDAVVDISHLSAFLVHEKAIETVV |
| CcpABCA1b-1 | MQCFLISTAFARANLAAACGGIIYFTLYLPYVLCVAWQDYVGFTAKVIASLLSPVAFGFGCEYFALFEEQGVGIQWNNLFSSPLEEDSYNLTTCLVLMYFDAFLYGVMTWYIEAVFPGQYGIPRPWYFPFTKSYWFGESNTNNTAVHGKKGNAWGLILKHVPAARMVEDLGHEITYVLPYKSAKDGAFVELFHDLDDRLADLCISSYGVSDTTLEEIFLKVAVDSGVDTEIISDGTVPVRRHRQHAFGVDHQSCLKPLTEDDNFDCNDSDGDPESKETDYLSVSNGKGSYQVKGWSLKRQQFVALLWKRFLYARRSRKGFFAQIVLPAVFVCIALVFSLIVPPFGKYPSLALNPWMYEEQFTFISDDAPQDTSTQKLLNALLDDPGFGTRCMEGQPIPDAPCTMGDEEWSTTEVAESVMDIFTSKNWTMDNPSPACECSCNGKKKMLPECPAGAGGLPPPQIKISETETLQNLTGRNISDYLVKTYAQIIGKSLKNKIWVNEFRYGGFSLGARSTQVLPPAEEIDDAISRVREIFQLGKDSGSAADRFLKSLSAFINGLDTKNNVKIWFNNKGWHSIGAYLNVMNNGILRASLPPGKDPRQFGITTFNHPLNLTKEQLSQVALMTTSVDVLVSICVIFAMSFVPASFVVFLIQERVTKAKHMQFISGVQPLLYWLANFLWDMCNYVVPATLVIVIFVCFQQKAYVSATNLPVLALLLLLYGWSITPLMYPASFLFKIPSTAYVVLTSVNILIGINGSVSTFVMDLFGNNEIGGINDILKNVLLIFPHFCLGRGLIDMVKNQAMADALERFGENRFRSPLEWDMVGKNLFAMAVEGVVFFIITVLIQYRFFFEPKLTIIGIVVSFTDVLYLGQVAEWGIHKLGLVKYVDKKAGSYSGGNMRKLSTAISLIGAPPVVFLDEPTTGMDPKARRALWNCIHSVIKEGRSVLLTSHSMEECEALCTRMAIMVNGRFRCLGSVQHLKN |
| CcpABCA1b-2 | SDTRANSTDVRLVAKATDSLLESLGSLAVEVDLILFTYHTTDKTATALASMRSWSDLRNEILFLTENATGSPSLMYQAVSRIVCGHPEGGGLKIKSLNWYEDSNFQALFGSNNDSDNEPVSVYDNTTTPYCNNLMKNMEASPISRMIWRALKPLLMGKILYTPHSPATQKIIHEVNKTFQELGILRDLGGMWEEARPKVWNFIENSEEMDLIRTLLRNNATASFFSAQLAGTQWSVEDVTSFLSKHSEDTRPHGTAFTWRDVFNETDQAIMSISRFMECVNLDKLEPVSTEEKMVNESMSLLDNRKFWAGIVFLDIQSNSSKLPPHVNYKIRMDIDNVERTNKIKDGYWDPGPRADPFEDLRYIWGGFTYLQDIVEHSIIRAVTGTKEKTGVYIQQMPYPCYVDDIFLRIMSRSMPLFMTLAWMYSVAIIIKGVVYEKEARLKETMRIMGLDNGILWFSWFISSLIPLLISAGLLVLLLKMGNLLPYSDPGVVFLFLGSFAVVTIMQCFSNQYSVRTCQPLYLPYVLCVAWQDYVGFSAKVIASLLSPVAFGFGCEYFALFEEQGVGIQWNNLFSSPMEEDNYNLTTCLILMYFDAFLYGVMTWYIEAVFPGQYGIPRPWYFPFTKSYWFGESNTNNTAVHGKRAMLEVAFGFGCEYFALFEEQGVGIQWNNLFSSPMEEDNYNLTTCLILMYFDAFLYGDDNASESSSDAGLGSDQESEAATAIGTTWPESPVVPVDVDLISSLILKHVPASRMVEDLGHEITYVLPYESAKDGAFVELFHDLDDRLADLGISSYGISDTTLEEIFLKVAEDSGVDTEIISDGTLPVPRHRRHAFGADHQSCLKPLTEDDDFDCNESDGDPESKETDYLSVSNGKGSYQVKGWSLKRQQFVALLWKRFLYARRSRKGFFAQIVLPAVFVCIALVFSLIVPPFGKYPSLALNPWMYEEQFTFISCNGKKKMLPECPAGAGGLPPPQIKMSETETLQNLTGRNISDYLVKTYAQIIGKSLKNKIWVNEFRYGGFSLGARSTQVLPPAEEIDDAISRVREIFQLEKDSGSAADRFLKSLSTFINGLDTKNNVKIWFNNKGWHSIGAFLNVMNNGILRANLPAGKDPRQFGITAFNHPLNLTKEQLSQVALMTTSVDVLVSICVIFAMSFVPASFVVFLIQERVNKAKHMQFISGVQPYLYWLANFLWDMCNYVVPATLVILIFVCFQQKAYVSATNLPVLALLLLLYGWSITPLMYPASFLFKIPSTAYVVLTSVNILIGINGSVSTFVMELFGNNVRKCFFGFILMQSYDLLAILSSNSQAHIVISNAAHPLLQEIGGINDILKNVLLIFPHFCLGRGLIDMVKNQAMADALERFGENRFRSPLEWDMVGKNLFAMAVEGVVFFIITVLIQYRFFFEPKLTLIETINVLFLGQVAEWGIRKLGLVKYVDKKAGSYSGGNMRKLSTAISLIGAPPVVFLDEPTTGMDPKARRALWNCIHSVIKEGRSVVLTSHSMEECEALCTRMAIMVNGRFRCLGSVQHLKNRFGDGYTIILKVAGPDPDLQPVMKFIESELPGSTLKEKHRNMLQYQLPSSLTSLAHIFSILAKNKEFLRIEDYSVSQTTLDQVFVNFAMDQSDDHFDSSIRRKETAVNMALLSPLSAKENTEKPIESFV |
| CcpABCA2 | LADVERLLRDVDLLSGLARLLPKGACAGHQPPPITNTTSWSSNTTTWGLNTTDSPVEEGDRAAGNEAEAENPRSQFSAFVQLWAGLQPILCGNNRIIEPEALKQGNMSSLGFTSKEQRNLGLLVHLMTTNPKILYSPIGSEVDKVIQKANETFAFVGNVTHYARVWLNISAELRAFLEEGVIFQTNKDGSLPPHVMYKIRQNSSFTEKTNEIRRAYWRPGPNTGGKFYFLYGFVWIQDMIERAIINTFVGHDVVEPGNYVQMFPYPCYTRDDFLFVIEHMMPLCMVISWVYSVAMMIQHIVAEKEQRLKEVMKMMGLNNAVHWVAWFITGFVQLSISVTALTVILKYGKVLLHSDPFIIWLFLTIYAVATIMFCFLVSVLYSKAKLASACGGIIYFLSYVPYMYVAIREEVAHDKITAFEKCIASLMSTTAFGLGSKYFALYEVAGVGIQWRTINQSPVEGDDFNLLLSMVMLTIDAIVYGVLTWYIEAVHPGMYGLPRPWYFPLQKSYWLGSGRIETWEWPWGGSTRLSVMEEDQACAMEHRRAGMYGLPRPWYFPLQKSYWLGSGRIETWEWPWGGSTRLSVMEEDQACAMEHRRAEETRGIEEEPSHLPLVVCIDKLTKVYKTGSKLDPKQLTQSSQSPASSISPCSESRVTQFIRQYVASCLLVSDSNTELSYVLPSEAVRKGCFERLFQALEQSLDSLALTSFGVMDTTLEEVFLKVSEEDLSQENSDADMKDSPGGASAGKPSNLLGGPQCEGAPAAAVIRPEVELSNLMMCSRLSPSQASLQSGSSLGSIRGDERGLYSDFYGDYCPLFDNGQDPDSASLRDEESSPERTIQEHQGSLKLEGWWLKLRQFHGLIVKRFHCAKRNTKGIFSQILLPAFFVCVAMTVALSVPEIGDLPPLILSPSQYHNYTQPRGNFIPYANEDRLQYRSKLSPDASPQRIVNTLRLPSGVGATCVLKTPFNSTLDQLAQSLNPYANNSKTLAARYFDSMCLDSFTQGVPLSNFVPPPPSPAPSDDPDAHFEDGLWNYTAAPPTTVREMATSPPTLPLTIREPVRCICSMQGTGFSCPSGVGGRPPLMKVVTGDILVDITGRNVSEYLLYTSDRLRLHRYGGFTVGNIQKSVPASFGRKTPPMVRKIAVRRSSQVLYNNKGYHSMPTYLNVLNNAILRANLPSSKGNPAAYGESELAAGITVTNHPMNRTSASLSLDYLLQGTDVVIAIFIIVAMSFVPASFVVFLVAEKSTKAKHLQFVSGCDPVTYWLANYIWDMLNYLVPATCCVLILFVFDLPAYTSPTNFPAVLSLFLLYGWSITPIMYPASFWFEVPSTAYVFLIVINLFIGITATVATFLLQLFEHDKVVFDWTIFNNVLTTFLGLGSCVAVYARSLNAFSSLQASLISILRELLHVQQSIGYCPQFDALFDDLTAREHLELYTRLRGIPWKDEERVVQWALEKLELSKYADKPAGTYSGGNKRKLSTAIALIGYPSLIFLDEPTTGMDPKARRFLWNLILDIIKTGRSVVLTSHSMEECEALCTRLGIMVNGRFKCLGSIQHLKNRFEKSQPLKCVTRECSVLLK |
| CcpABCA3b | LFPPSSGRAYINGYDICQDMALIRRSLGLCPQHDVLFDNLTVREHLLFYTQLKGYPREKIPDEVDRTLRILNLEDKRHARSDLVFLCRFELLFAELEMNRDELGIASYGASVTTMEEVFLRVGKLVDSSLDIQAIQLPALQYQHERRSHDWTMDDSSSISGMTDVTDFTDSGTMISEDGSNIKLNIGARLYMQQFYAMFLKRALYSWRNWKVMVAQFLVPLIFTVLALVVARSLPGSKITPQLRLALKQYGPTHVPVAVDVSAGPLATALAEIYAAQLPSQNAIPATNITDLSEYVLYNAMREGGAFNEHCVVGATFRGRSRKNTEVIGYFNNQGYHTPATALMLVDNALYKLLAGPKASIQTGNYPLPRNMSETAQSQLSEGQTGFAIAINLMYGMASLASTFALLLVSERSVKSKHVQQVSGVYLSNFWFSALLWDLINFLLPCLLMLVVFRVFSVKAFVAENHLVDVLLLLLLYGWAVIPLMYLLSFMFSSPATAYTRLTIFNILSGTATFLAVTIMTIPELKLVDLSHLLDKIFLIFPNYCLGMSFSEFYQNYEIITFCTSSFFAEDICKYFNITYQVNYFSMDEPGVGRFLVAMSLQGVVFIALLFFIELRCIHVLFNLSMGPKKILLLAEEALQPEDRDVAEERKRVLECQPVVDSMVGSPLILQELSKV |
| CcpABCA4a | MGGKVLNYSNPIILFLFLLTFTTATIMQCFLMSVFFNKANLAAACSGIIYFTLYLPHIVCFAWQDRITKDMKIMASLMSQVAFGFGTEYLSRYEEQGLGLQWDNIQTSLLEGDEFSFLISIIMMVLDTVLYGILAWYLDNVFPGQYGIGRPFYFPIQPSYWLNRPEPLKQVPGKPAVEKPAFDNLVNHTDEKAKNHTAPEKPPEPPGSCKHKDIREKLEKEKELKRVDEEETTNKSMSTDSSGRTVILSTHHMDEADLLSDRVAIISQGRLYCCGSPLFLKNCLGAGFYLTLVQRIKEQPSHVRKEDTDRLVYANGQTNSASSTSKDGRGSHQVKGIFLVLKQFFALVIKRFHHTTRSGKDFLAQIVLPASFVLVSLMFTLIVPPFGEYPSLTLSPWIYGRQFTFFSNEQMQSSDMRYFGEVLLNRPGFGTRCMVDEPLEYVYMHSNDIKEYLKMFVLLTMSVFVSHRDYPCNNITTEWEMPLVNPALIEMLGNPEWTDISPSPSCQCSTPNKLTMLPVCPEGAGGLPPPQRIQSTGDVLMDLTGRNISDYLVKTYPNLIRTSLKSKYWVNEQRYGGISVGGLLPVLDVDPKAIQNAAAQLGHLLNVTGGRYSKVTLQEFGTFLRYMETENNVKVWFNNKGWHAMVAFMNVANNAILRANLPPGADLNEYGITAINHPLNLTKEQLSEVTVLTTSVDAVVAICVIFAMSFVPASFVLYLIQERVTQAKHLQFVSGVSPLVYWIANFFWDMINYAVSAAMVVGIFIAFDKKCYTSPGNLQALIALLMLYGWSVTPMMYPMSYVFNVPSTAYVSLSCINLFIGINSSAITFILDLFDSTEALYKCNQVLKKALLVFPHFCLGRGLIDMAMNRAVMDVYARFGEDFSLDPFSWDFVGKNVMFMVIEGFVYFILNVLIQYRFFLDHWMSQPQEWILTLGGSCGTPS |
| CcpABCA4b | MDFLDRRLKETQFSTKDILNFLHNGPEQDRYMNMTDFDWRNVFRLADDAIRMFNQYSEKRNEGLMGNPFFEPEPEGLIMGVSVQDLVKVYSKSSRPAVDCFNMNFYEGQITSFLGHNGAGKTTTLCVLTFSLSCDINDMKSFLFCVIWTYFSHVYLHCCRSILTGMFPPTSGTAYIYGKDIRTEMDAIRQSLGMCPQYNILFHHLTVEEHILFYSLLKGRDRKDAEQEVENMLEDLGLPHKRDEEAQNLSGGMQRKLSVAMAFVGGSKVVILDEPTSGVDPYSRRSIWDLLLKYRTGRTVILSTHHMDEADLLSDRVAIISKGKLHCSGSPLFLKNCFGVGFYLTLVRRMKDQRKKENECDCASQCSCTCSTCTRCKEESQAFQPERILDGNVESITTLIHHHVPEAKLIEMIGQEMTYLLPNKGFKYRAYASLFRELEETLGDMGLSSFGISDTSLEEIFLKVTADGEAANSSVTPEQWMLQQRKNGNGFLRDNEAASNVEDANGVGSPLESDNSAGRASRQVKGFSLVLKQFHALLVKRFHHAARSHKDFLAQIVLPASFVLIALVFTMIVPPFGEFPSLTLTPWMYGPQFTFISNEQPSHPKMRHFIQTLLKEPGMGTRCMANQPLESLFTCLNTTSDWEVPPVSPEVENILLSPEWDTRNPSPSCECSTDTKLTMLPVCPAGAGGLPPRQRKEPTGDILLDMTNKNISDYLVKTYPKLIKTSLKSKYWVNEQRYGGLSVGGHLPILDVDPEEIRALFSQLGRMMNITGGPYSKSVMNELGTFLHYMESEYNVKVWYNNKGWHAMVSFMNVANNAILRAYLPPHANPSEYCITAINHPLNLTKEQLSEVTVLTTSVDAVVAICVIFAMSFIPASFILYLIQERVTKAKHLQFVSGVSPLVYWVANFFWDMMNYSVSTAMVVGIFVGFDKKCYTSPTNLPALVALLCLYGWSVTPMMYPMSYMFNVPSTAYVSLSCINLFIGINSSAITFILELFENNRSLLMFNEVLKKVLLVFPHFCLGRGLIDMAMNQAVTDVYARFGEGVFYGSIQMELCGEESLLYGCGGVSYYGKSPVKDEDEDVAQER |
| CcpABCA5-1 | MQPITRRDAGVWHQTRSLLYKNLLIKWRTKQQSLQELILPLLLLGLLILVSTINPHVSYGSISNKELEYERNLSLKALGYTPINNVTNHIMEEVARELSMNEQLEMFSTEEQLDNASLYDPDGFVGVVFLDSMEYLLRFPFGQVPLPIDFTESITSCYTNYVNCHATNYWYSGFIRLQSLIDAAIIQMKTKRPVWNELKVRAVMMGHPGTVEVQRSHYALISIYLVLAFTPFVSFLIVNVVTEKEQRLKDTMGMMGLYDSAFWLSWGLLYAALVTTMSILMAVIATCTPLFSKSNFFVIFLLIFLYGISSCFLYRHRPGEFGTRRSVLYFLQPSYWSKRYVKVSSVYEGEVNGTPVNDESVEAVSPEFRGKEVIRCRLETQIDQWSTIWLFAELDCRPDLGIINYGVSMTTLEDVFLRLEAEAEVDQSDYSVFNQEKEEDEGDASSMDDTDQRLLMFSDSRQDAVTGRALWRQQFSTVAWLHMVNLKEREKIYHKQVSTC |
| CcpABCA5-2 | MCQSAEACWKNAAYNQKRCRSLASDEEPALQKPADQMEDQTTESPAVSDPCASQLKHAGRMQPITRRDAGVWHQTRSLLYKNLLIKWRTKQQSLQELILPLLLLGLLILISTLNPHVSYGSISTKELEYERDLSIKGLGYTPINNVTNHIMEEVARELSMNERLEMFSTEKDLENASLYEPDGFVGVVFLDSVSYRLRFPYNQLPLPSDFTESITSCYTNYVNCRAANYWYSGFIRLQSLIDAAIIQMKTKRPVWNELKVRAVMMGHPGSVEVQKFPHALISIYLVLAFTPFVSFLIVNVAAEKEQRLKDTMGMMGLYDSAFWLSWGLLYAALVTTMSILMAVIATCTPLFSNSNFFVIFLLIFLYGISSIFFSFMLTPLFKKPKFASTVGSMLTVVFGCLSLFTVLMRDFPQSAVWLLCLLSPSAFSIGIAQVVYLEAQGDGAVFSSLGNGPHALYVPMVMLFLDCILYLLLAIYLDQVLPGEFGTRRSVLYFLQPSYWSRRRKRYVEVSSVYEGEVNGTPVNDESVEAVSPEFRGKEVISTKNIPYISMLDCRPDLGIINYGVSMTTLEDVFLRLEAEAEVDQADQLFNILLFDINYVKLISITVNQHSELLKRYNTHSGFPSLVDYSVFNQEKLEDEGDVSSIDETDQRLLTFSDNRQDAVTGGALWRQQFSTVAWLHMLNLQRERKPFIYNLTLFLVFLSAMLILSVATGNIQIHSPERLFSPIYLLHRNEAPRKYTTSLLVQNSTDSDLSGFIHNLASQDIKVEMMKKPDYMSAAPVSAAINVTGSSKDFSYILAFNSTTVHSLPMVVNVLSNALLRGFNGTEHIKTWTKPFDYQIPDKTSYALVYIEAVILGMLAAGMPAYFAMDHTRDKELTCRSTMRISGLVPSAYWCGQAAIDVPFFYLILICMTSTLFAFHSTNLLTSYNIMSVALCLIGFAPAMVLFTYCVSFMFVKVQSNRDFFSVVSMMLCVVSASIVQMALVNENAGMARLLHNALCFFSPLYPLMGCLNCITMATFLQSPHEEDFPWKNLFISVVSPYIQCILMLFTLRWLEIRYGGKTMKNDQICSVGVLCFALSMLGNPQIVLLDEPSTGMDPKSKQRM |
| CcpABCA12 | MHKKCALHLLISDLSTPKVSQQHRFCLPNGSHDFLGAVCRPLCEKLVHERELRLHEYMKMMGVNPISHFFAWLIESAVFLLATIIILTIILKAGGILPRSDGFVLFLYLCDYGFSVLAISFLVSSFFDKTNIAGLSGSLIYVICFFPFIVLIHLEDNLSFSVKSALSLFSPTCFCYASQYISRYEKQEEGIQWSNMYISPLAGDTSSFGWLCWLLLIDSVVYFIIGIYIRMVFPGKYGIAVPWYFPVTRSFWTDVFSCCNRTPKKIGRGLLFSNMMHEQKNTDKSKEHTIILSTHHLDEAEVLSDRIAFLERGGLKCCGSPFYLKDKLAKGYNLTLTKKVQTPDSNEKFNIEELRTFIQSYLPDARQKEGEVGDLVYALPPYTPQNAAVYHSLLTGLDQNLDKLQLGCYGISDTTLEEVFLQLTRDDLEPKESETWSVSESVMENDMFASRDSIPDDFNNTYLGEKASLTGTSAVRGFTLTAQRVMAMLLKRVHHSRRDWKGLFSQVLLPVLFVIAAMGLGSIKSDLQHFPEIVLSPALYHVDEQYSFFSNQNPTTNSLVDSMMSYPGIDHVCMRDPTNSVCKERPTVGSENWISRGNSSATFTTCKCSNQVQSCPASDHEPPHKRNPSSQIVYNLTGINTEDYLLATANDFIRIRYGGWDFGKPLPIDLKMDMLDVPANRTLSKVWYNPEGHHTMPAYLNSLNNFILRSNLPLEKRQQYAISISSHPYPGQVQDEDVMVGGLVSILVALCVLTGYSIMTASFVIYEVQEHHTGSKRLQQISGISEPFYWIINFFYDMALYLVPVVLSVAMIAAFQLSAFTDRQNLGAVTLLLVLFGFSTFPWMYLLSAVFKDTEMAFIGYVCINLFISVNTIISTSIIYFLGQLNQNDQSILNVYHTMSNIFLVFPQFSFGNGLMELARVDMQVQILSAFGVDAYKNPFSMDVLGWMYISMFLQGFICFTLRLLLNKTLLRKVRLLVCWKKNVVQSYSPNEDEDVVAERLRVDRGDANADILQVNHLTKVYQNLSKRVQAVKRLSVGIPAGEVVNYLLKKLELNYHRHNTSESYSCGTRRKLSTALALIGNPQILLLDEPSSGMDPRSKRHLWKIISEQVMGKCAVVLTSHSMEECEALCTRLAIM |
| CcpABCB4 | MGKKDKLNISNGKKEENGDVSGEKNGKEEEKEKVEMVGPFEVFRYADGVDIFLMLVGTVMSIANGAVLPLMVIVFGGMTNSFVDDTIAENLKNITLSPNFTFPQSSNETLGEQMTRHAIYYSIMGFVVLFAAYMQVAFWTLAAGRQVKKLRKRFFHSIMKQEIGWFDVNETGQLNTRLTDDVYKINEGIGDKLGMLLQNLTTFITGIIIGFAHGWKLTLVILAVTPFTDVYKINEGIGDKLGMLLQNLTTFITGIIIGFAHGWKLTLVILAVSPLLGISAAIIAKVMTSFTSREQTAYAKAGAVAEEVLSSIRTVFAFGGQKKEILRYHKNLEDAKNVGIRKAVTVNIAMGFTFFMIYMSYALAFWYGSTLILAGEYNIGILLTVFFSVLIGAFGIGQTSPNIQSFSSARGAAHKVFHIIDHMFKSTEDTEEGKEEMTMDEKSPSVSSLNDRALFRQKSRSGSEKDQQDEEKQTEEENAPNISFLTVLKLNQPEWPYMVVGVLCATINGGLQPAFAVIFSKIIAVFAEIDQDLVRKRTELYSLLFAGIGVVSFFTLFFQGFCFGKAGEILTMRLRFKAFNAMMRQDLSWYDNTKNSVGALTTRLAADTAQVQGATGVRLATLAQNVANLGTAIIISFVYGWQLTLLILSIVPIMAVAGAIQMKLLAGHALKDKKELEQAGKIATEAIENIRTVVSLTRESKFETLYEENLVVPYENAKKKAHVFGLTFSFSQAMIYFAYAGCFKFGSWLIEQKLMTFEGVFLVISAVVYGAMAVGEANSFTPNYAKAKMAASHVLMLINRVPAIDNASEDGDKPVFLSHMHLNTRIQSMMGLSLNNNVQ |
| CcpABCB5-1 | MKEEPSVQSTDPPPYLQEVIPEGFVNLAYTQDEKPQEDKPEETSAKSKGKGKKEKKYKKEDRASENSWVFSAAAHGVALPLMCVVFGEMTDSFVLSGHTANLTGNFSGNFTYALTNSSVCLAGSPEIGIEDKMSKHAYYFVAIGAAVLLLGTFQVMLFLLTAAKQTKRIREKYFHAILHQQMSWFDTHQIGELNIRLTEYVKCSHVSDSCFCHLISSVVTSQFFCTFISGFIIGFIYGWKLTLVILAVSPLLAGSAAVWSKILATLTSKELTAYAKAGAVAEEILVAIRTVVAFNGQKKAVEKGDKIGPKCQECDQAVLNVSIKDEWFSVTNYFLYSRYEKNLIEAKNFGVKKAITTNVSMGLTQFIIFGTYALAFWYGTKLSVDEPENYSIGRVIIVFFSVMIGSFSLGQGAPNLESIAKARGAAYEVYKTIDMPRPIDSSSNEGHKPDHVIGDIEFKNIHFSYPSRKDVKGQKALGFHQKIYLCPKMNKGFTGLEQHETCGRPNDDDLDANEDDTQDASEGETCEESSETETPAEGMEMQMESGTLRRSLRRGSERRSSRKKSSKKKSKKSKQDKKEKEKAPEIPFTKILALNKPEWPYLLVGTLASLVGGAVYPCVAILFAKIIGVFAEVDPDVKRQKTMMFSLFFLLTGAVAFITYFFQGFMFGKSGELLTMRLRRQAFNAMMRQPVSLNRRLLGLMTTTMTVGVLTTKLATDASLVKGISTETVENFRTVVALTREDVFFHKFIDSLSKPYQ |
| CcpABCB5-2 | MAKESRPMTAFVTPWGLYEWIRNPYGLMNAPAAFQHCMEECLEDLRDNYCVPYLDDTLLFSKSFEDHVNDVRKVLQHLRRYGIKLKPRGQHKHCAITMKEELSVQSTDPPPYLQEVIPEGFTNLAYTQDEKPQEDKPDEPPKESSEKSKCKGIKAKKSKKKKEPVKTVGFFQLFRYATCLEVLLMLIGLLCAAAHGIALPLMCVVFGQMTDSFVLSGQKGNLTGNFTENFTFINSSTCLAGSPEIGIEDKMTEHSYYFIAIGAAVLILGTFQILASLTSKELTAYAKAGAVAEEILFAFRTVVAFNGQKKAVEKYEKNLVEAKNFGVFFSVMIGAFSLGQGAPNLESIATARGAAYEVYKTIDMTSGRPNDDLDGNEYDIQDVSEGETSEESSETETPEGGMERQMESGTLRRSLRRGSERRSSRKKSSAKKKSKKSKKDKKEKENVSEIPFTKILALNKLEWPYLLVGTLASLVGGAVYPCVAILFAKIVGVFAEVDPEVKRQKTMIFSLFFLLTGAVAFITYFFQCPQEIALLQGAAVSGESFSQLPPGNVVVLRGLLPLRRSLEQLVWPSPAGSPLQGTDLGALITPVVSLKRLISLVDYLTAWKLLPNVSRWVLHTVERGYRIQRGSLPPSVQRGDSHSGGPREGSGHLHTQPHRRLVDIRSIVLAHMKEGLRHNAKKSVLSPVQRTTYLGMVWDLTTKQTRLSPAWIESILTAVAIVREGRSLTVKLFQKLLGLMAAASNVIPFGLLCMRPLQWWLKTKDLGMLKIMRRCLCPLDMWRKPCFLSQGPGFMFGKSGELLTMRLRSQAFNAMMRQEIAWFDDDNNAVGVLTTKLATDASLVKGAITEQPALLVSAMEDITEPPNFTAAAIVSKAVFTFHLRGVLRAWTASESTQESATVHVSAPEPTPTHESAQLWWSRALPTLPWWSSAPPTPMWWASAPSALPWMVSALPAPYWRASALHVPPWRASVPHVSPHGPGRPSRPLDRLRSTWGAGMAGDWGASGEHLESAPTMFIGWARLSLLLVHFFLPHLFLPFASLLICLDTELCEQPASFAMTFCEGVNGRLSIKFDTSGFYGSYDKENMELILK |
| CcpABCB9 | MGTLTVRENLRFSAALRLPKSICQQEKDEKIEKLIQELGLSKVADSRGEPALICEKLGPANSGVQWQMPIELHGARQMANSSRTIILSIHQPRYSIYRLFDSLTLLLGGRLVYHGPAQDALDYFSQIGYICEPHNNPADFFLDVINGESTAVALNKLYDIEELDQEQLRSSLKGIEDRLVEEYKTSTSNKQNQKRAGANHSGKGLQQTAQIQNHHLQHILLPPVQLGPQENLQGPHAEPTDLIGSGQHQRLHQAFTDVLKAFELK |
| CcpABCB11a | MVGFIGGWKLTLVVIASVARLTGRELKAYAKAGAVADEVLSSLRTVAAFGGEHKEAERYDRNLVEAQAWGIRRERSLVCFRVTFGASYSCVTLWLSGDHLEHLTPETNFTEEDGFEKVRSFSRGSYESALRRSLRQRTHSQMSNNIPDAISGKFEIHSDQFEIEEEETNNTKSKGEDKEVIEAAPVARILKYNRPEWPYMLLGSLGAAINGSVNPIYALLFSEILGTFSIQDLDEQRRQINGICILFVSIGVMSFFSQFLQGYSFAKSGELLTRRLRKVAFQAMLKQEIGWFDNPFNSPGALTTRLATDASMVQGATGSQIGMIVNSLTNIGASFIIAYYFSWKLSLVVTCFLPLIGLSGVFQAKMLTGFANEDKNALEAAGQVSSEALSNIRTIAGLAKEKHFVSQYEEQLQTPYKGAKKKAHVYGICFAFAQCVIFMAFAASFSIRWLSGQ |
| CcpABCB11b | MGRRLGDAAKKAISQLQVCTIRKGDQETESDFDNCAVCIEGYKPNDVVRILPCRLLDIEYEMTNFAYYYVGIGAGVFILGYLQISLWITAAARQIQIIRKMYFRKVMRMEIGCDINKINDAIADQVGIFIQRFTTFVCGFLMGFARGWKLTLVIISVSPLIGVGAGLMALFVAKLTGLELQAYAKAGAVADEVLSSVRTVAAFGGEKKEVQRYDRNLISAQRWGIRKGLIMGFFTGYLWFIIFLCYALAFWYGSSLVVDTQEYSPGTLLQVFFGVLIAALNLGQASPCLEAFASGRGAATIIFETIDRRYINKEMEEAESSDPVAERKSLNRAGSYRASLRASVHQRSRSQLSNLVPESSVAIAGELGPRSYSETRVPQEIKGKSGLPDDDSEEVEPAPVARILKYNAPEWPYMLFGSFGAAVNGGVNPVYSLLFSQILAGYAFSKSGELLTRRLRRLGFHAMLGQEIGWFDDHRNSPGALTTRLATDASQVQGATGSQIGMIVNSLTNIGVAIIISFYFSWKLTLVILCFLPFLALSGGFQAKMLTGFAKQDKDAMEAAGQISGEALNNIRTIAGLGKERNFVEMFEVQLEAPYQAALKKANVYGACYGFAQCVVFMANSASYRFGGYLVYREGLHFSFVFRICYSVHAVCSSDDLDVCVDSSAVCACGFVGSSALFVEIGEHLDVAPSDPAMQLLETVSARITRLSRCRSAGRSPHADPRRINRRSHETSRRNPNRWPASSGTHGAEMAIKQRGPVSAVLTDSSVSKRCDHDLQLTTSQWRRSEDVVNVLKPMITLIELLSQDMNTSLSATVPMLMNIKKRHLVVHEDDSAVTKTLKPTLTEEIDR |
| CcpABCC1 | LMVTGYKRPLEEKDLWSLNTEDKSQRVVPQLALVITNAARRTSTVGEIVNLMSVDAQRFMDLITYINMIWSAPLQVILALYFLWQNLGPSVLAGVAVMVLMVPLNAVICHEKETYQVAQMKSKDNRIKLMNEVLNGIKVLKLYAWELAFKDKVSAIRESELRVLMKTAYLGAVSTFTWVCAPFLVALSTFAVYVLVDEHNILDAQKAFVSLALFNILRFPLNMLPMVISSMVQASVSMKRLRVFLSHEELDEDNVERPAISSSPDSIRIVEGAFSWSKDDSPTLKRQSLNSLNTAGTGKTPQKTEPNDVAASKKTKSADAARLTEADKANTGRKRSALKKKALRNLYKPGSIAVFCYSVTVSVGIAVFCYSVAVSVGGILASRYLHQTMLYNVLRSPMSFFERTPSGNLVNRFAKETDTIDSVIPSIIKMFMGSMFNVLGSCAVILIATPLAEWRLEHSTLPAGWPTAGHIEIHNFGLRYREDLELAISDISVNIEGGEKDPVLFSGSLRMNLDPFDGYTDEEVWRALELAHLKNFVSGLPDKLNHECSEGGENL |
| CcpABCC2-1 | MSAALEEYCGSVFWNASYLQRPDPDLPICVEQTVLVWFPLAFLWLCAPWHFANLCKKSAKAPLSKLYICKQVVAGLLLLTAIAELALTLGEDYGPSSDSTVQNHPAVLYTNPVLFAVSWVVCAVFQFQSLLRDALNKFQSLLRDALNKEEIADLPRFCLFYISYGLQLIALVLSAIADVSPEVKRISQMNPEARATFLSRITFNWFNSMVIKGYKRPLVQEDMWDLNEKDSSQAFCQGFEEVMAKELIKARCSLQKKQNKRKTKSDHQNGLAKGVSQDVLVMTRGGDRKEERKKKKKKDSESEYPNSWLVPTIAKTSKAVLLESAFYKLIQDLLSFASPQLLKLMISFTQDKSSHAWTGYLYAVLLLVVAFLQSVILQQYFQRCFILGMKVRTSLMAAVYKKALVVSNDSRKESTAGEIVNLMSADAQRFNDVTNFIHLLWSCPLQIALAIAFLWIELGPSVLAGLLVMVLMVPINGWLATKSRGFQMENMKFKDKRMKIINDILNGIKVLKYYAWESSFEAQVQEIREKELKVMRKFAYLSSVSTFIFSCAPAIVSLATFAVFVSVSPDNILDAEKAFTSISLFNILRFPLAMLPQLISIMVLESDFEQIEMLPEGLEPQADGCPEDIVSSTLKRENSLRHSQRHSKQNGSMRLRKNSSVRSKKDSGDKKGQRLIEKETMETGRVKFSVYLQYLRSMGWCFITWSFLFYFIQNVAFIGQNLWLSDWTEDSVEYFNTTYPNHIRDMRIGVFGALGLAQGFLVFLGTILLADGSISASRTLHTSLLSNILKVPMVFFDTTPSGRIVNRFAKDIFTVDEIIPMSFRSCILCLLGVLGTLFVICLATPIFTAVVVPIAVIYYFVQRFYVATSRQLRRLDSVSRSPIYSHFGETVSGISVIRAYGHQQRFLKQNEDTIDQNLKSVYPWIVSNRWLAMRLEFLGNLVVFFSALFAVISRDSLNSGLVGLSISYALNVTQTLNWLVRMTSELETNIVAVERVREYAEIKMRVMVLDSGKIVEFDSPSVLLNNKQGHFYAMAKDAGIRGGETSKPTDISTDL |
| CcpABCC2-2 | MSAALEEYCGSIFWNASYLQRSDPDLPICVERTVLVWLPLAFLWLCAPWHFATLCRKPAKAPLSKLYICKQVVAGLLLLTAIAELALTLGEDYGPSSDSTAQKHPAVLYTNPVLFAVSWMVVMLCQECVRRRPRCVDSGSLFVFWLLQVLCAVFQFQTLLRDALNKEEITDLPRFCLFYISYGLQLIALVLSAIADVSPEVKRISQTNPEAKATFLSRITFNWFNSMVIKGFKRPLVQEDMWDLNEKDSTQTICQEFEDIMTKELKKAHSRLQKKQNKPKTKTDHQNGLAKGVSQDVLVMEEMGKKKEKKKKKKDSESNYPNSWLVPTIAKTFKAAGSQDTTSHAWTGYLYAVLLLVVAFLQSVFLQQYFQRCFILGMKVRTALMAAVYKKALVVSNDSRKESTAGEIVNLMSADAQRFNDVTNFIHLLWSCPLQIILAIAFLWIELGPSVLAGLLTMVLLVPINGWLATKSRGFQVENMKFKDKRMKIVNDILNGIKVLKYYAWESSFEAQVQEIREKELKVMRKFAYLSSVSTFIFSCAPAIVSLATFAVFVSVSPDNILDAEKAFTSISLFNILRFPLAMLPQLISTMVQTTVSKKRLEKFLSGDDLDTMAVTRDDSHRSYDGLRASKGAFSEFLETYGKEESNNADARKALAQESDYEQIDTLPEGMEPQADDSPEDIVSSTLKRENSLRHSKRNGSVRVRKNSSVRSKKDSGDKKGQRLIEKEAMETGRVKFSVYLQYLRSMGWCFVTWSFLFYFIQNVAVIGQNLWLSDWTEDSVKYFNTTYPTHIRDMRIGVFGALGLAQGFLVFLGTILLADGSISASRTLHTNLLSNILKVPMVFFDTTPSGRIVNRFAKDIFTVDEMIPMSFRSWILCLLGVLGTLFIICLATPIFTAVIVPMAIVYYFVQRFYVATSRQLRRLDSVSRSPIYSHFGETVSGLSVIRAYGHQKRFLKQNEDTIDQNLKSVYPWIVSNRWLAMRLESLGNLVVFFAALFAVISRDSLKSGLVGLSISYALNVTQTLNWLVRMTSELETNIVAVERVREYAEIENEGNGPGFWVMVLDSVGGADSWKEEAANKKLKKKKQKAKKSMGRTSNTTQAFLGPANSFAPVAAEERTDHRRWTCVSQDGVDPKRGERSSAGPLAGGVQRAGAGRRGPGRGYQVETHVH |
| CcpABCC4-1 | WLNPLFRIGSKRRLEEDDMYKVLPEDGSKRLGEELQSWLNPLFRIGSKRRLEEDDMYKVLPEDGSKRLGEELQSWLNPLFRIGSKRRLEEDDMYKVLPEDGSKRLGEELQRSRTLGGGYREQGVRESKSYWDQEVEKATKELRTPKLTKAMIRCYWRSYAVLGVFTLIEEMIKVIQPVFLGKLILYFESYNPDNAELYEAYGYAAGVSLSTLGLALLHHLYFYHVQRAGMKIRIAMCHMIYRKALCLSATAMGQTTTGQIVNLLSNDVNKFDEVTIFLHFLWVAPLQAAAVIGLLWQEIGASCLAGMAVLFFLMPLQTIFGKLFSKYRSKTAAFTDSRIRTMNEVVSGIRIIKMYAWEKPFAALVNDVRRKEISKIMSSSYLRGLNMASFFAANKIILFVTFTVYVLVGYKISASRVFVAVSLYSAVRLTVTLFFPAAIEKVSESAISIRRIKKFLLLDELVKAHVPLTQEDKKEASVEIQDLICYWDKTLDAPTLQNVSFTVKPGQLLAVIGPVGAGKSSLLSTVLEELPAEKGVIKVKGELTYASQQPWVFPGAVDAEVGRHLFEQCICGILKEKPRILVTHQLQYLKAADQILVLKEGHMVARGTYSELQQSGVDFTSLLKKDEEEEGEKEEAPRSPRNRTISQNSVRSHSSSVLSVKDESDQLPAEPVHTMAEETRTEGTIGLRMYWKYFRAGANVIMLILFVLLNLLAQAFYILHDWWLSYWATEQRKLDFHSTNITANGTNGTNTNQELNLDFYLGVYAGMLMIEVRKSTNVDMLHVPPNGNGDLCILCSSGLTGATIIFGFMRSLFMFNALVSSAETLHNRMFNSILRTPVRFFDINPIGRILNRFAKDIGHLDSLLPWTFVDFIQVFLQIIGVVAVASSVIPWILIPVLPLLISFLFLRRYFLRTSRDVKRIESTTRSPVFSHLSSSLQGLCTIRAFKAEERFQQTFDAHQDLHSEAWFLFLTTSRWFAVRLDGMCSVFVTITAFGCLLLKDNQFVKRLCLVLTGMKAGDVGLALSYAVTLMGMFQWGVRQSAEVENMVLDAGRIQEYDAPHVLLQNQNGIFYKMVQQTGKAEVASLLQTAKQVSLKGHTLLVKKFGVIRIFNVLERSLLCSSRLHLFDQKYSVHEPQSSPPAQRLRHHRRWQLDYFRDCFITAREVPDVHSAQLRPATDTSSASSPQNSRLSGAGDAYMCSTLNVHTREDRAAITPEDFIHQPSDFSSMTAIG |
| CcpABCC4-2 | MAALYEAYGYAAGISLSTLGLALLHHLYFYHVQRAGMKIRIAMCHMIYKKALCLSATAMGQTTTGQIVNLLSNDVNKFDELTIFLHFLWVGPLQAAAVIGLLWQEIGASCLAGMAVLIFLMPLQTMFGKLFSKYRSKTAAFTDSRIRTMNEVVSGIRIIKMYAWEKPFAALVNDVRRKEISKIMSSSYLRGLNMASFFTANKIILFVTFTVYVLVGNKISASRVFVTVSLYSAVRLTVTLFFPSAIEKVSESAISIRRIKKFLLLDELVKSHVPLTQEDRKEASVEIEDLICYWDKSLDAPTLQNLSLTVKPGQLLAVIGPVGAGKSSLLSTVLGELPAEKGVVKVKGELTYASQQPWVFPGTIRSNILFGKELQPQRYESVLRACALKRDADIYLLDDPLSAVDAEVGRHLFEQCICGILKDKPRILVTHQLQYLKAANQILVLKEGHMVARGTYSELQRSGVDFTSLLKKDEEEECEKGEAPRSPRSRTLSQNSIRSHSSSVLSVKDESDQLPAEPVHTIAEETRIEGTIGLHMYWKYFRAGANVLILILLVLLNLLAQTFYILHDWWLSYWATEQEKLDFSSSNISSTIGRNGTFGTNTTQELNLDFYLGIYAGLTGATIIFGFMRGLIMFNVLVNSTETLHNRMFNSILRTPVHFFDVNPIGESLTGSPKTSANLIPYFHGHLWILSRCASTLLR |
| CcpABCC5-1 | MVELRHMSQCVAARWVRTVIRRVYKDAFETAARAEGGLSLDGGSASDAKELEDEVGRSKYHHSVCVLKPIRSTSKYQHPVDNAGLFSFMTFNWLTSLVVLAHKKGQLFLEDIWAVSQFESCEANRRRLAGLWDEEVRSRGDGASLRRVVWHFCRTRLLLSILCLMVTQLAGFSGPVPGCPFNLRGEHTAGRAGEDSWEAFVVRRLLEYTQMSEPDLPYGLLLVLGLLATELIRSWSLALTWALNYRTGTRLRGAILTMAFHKILRLRSLREKSMGELINMCSSDGQRMFEAAAVGSLLAGGPLVAVLGMAYNVSVLGPTSLLGSAVFILFYPTMMFSSRLTAYFRRKGVAVTDQRVQKMNEILNYIKFIKMYAWVKAFSQAVRRIRDEERQILEQTGYFQSITVGVAPIVVVIASVATFSTHMLLGYDLTAAQAFTVVTVFNAMTFALKVTPFSVKSLSEASVAIDRFKSLFMMAEVKMIRELPSSPSIAIEMTGASLAWETGGHSAQPSPRGTPYVGLGMRGCRKKRRQRDDPKHHGMLEEETRGQLLNDVPVDMASIPDEQTLQVPTISQRLQRTLHCIDLSIQKGKLVGVCGSVGSGKTSLISAILGQMTLLEGTVAVNGDFAYVAQQAWILNASFRDNILFGKEWRKRDDSSRGDCCCKWRFRICGHNRPGFSMHPSVTISCLARKWRKRDVQYSFRRYQAILSACCLRPDLAILPNADLTEVPNKNSGSSLKKPLESKKAGSVKKEKRPTRVMVSQLMQVEERGKGSVPWAVYRVYIQALGGWPVFLFILALFVLNVGSTAFSNWWLSYWIKQGSGNTTVQVGNSSVLSESMRDNPLMQHYAAVYAMSMGIMLLLKLLRGIVFVKGTLRASSRLHDDLFQKILRSPMKFFDTTPTARILNRFSKDMDEVDTRLPFQAEMFIQNVLLVLFCLGVIASVFPCIQGLTTTRLPFQAEMFIQNVLLVLFCLGVIASVFPWFLVAVGPLVLLFIVLHVISRVFIRELKRLDNITQSPFLSHIASSIQGLTTVHAYGKEDEFLHRYQELLDQNQAPFYLFSCAMRWLAVRLDVISVALISITALMIVLMHGKIPPAYAGLAISYAVQ |
| CcpABCC5-2 | MTLQWLSPLAWRAHKESCLKIEDVWGLSCHEASETNCQRLEWLWHEELKRRGKDVASLSRVFWRFCQTRMLVAIFSLLITMVAGFVGPALLIRALLEYSQCAELVNICSSDGQRLYEAVSVGCLLAGGPLVGILGLSYTIYFFGPTALVGSAIFVIFYPTMMLASRLTAYFRKRCVTVTDRRVRLMNEILGCIKFIKMYCWETAFASNIQRVRSEERRVLEWAGCVQSLTVGVAPVVVVIASVCTFTLHMALGYDLTAAQAFTVVAVFNSMTFALKVTPLAVRALSEGSVAVKRFQKLFLMEDREPISSKTEDPYNAVEFKDATLAWEKTCSSQGEKSRAQQTRGGMKRVLRREKLSLHITTEDSKEESEEANAEHLLTHMEQESPQSTISSTQSIRPPLHKTLHRIDLCIRKMTLLGGSVAVNGDFAYVAQQAWILNDSLRENILFGKKYIEEKYNAVLEACCLFPDIIELPYGDMTEFLPECDEVVLMKDGQIAEHGTHVQLMEKGRDYAALFNSVQQENLVRKNVKNKQRAEEESSPQSLHVSPAAKPHTESKKGDQLMQAEEKGSGAVAWQVYTTYIKAAGGPLAFIVNILLFLFTTGSIAFSNWWLSHWIRQGSG |
| CcpABCC6a | MGLSPDIVRYLAFFSYFALQLAQLFLSCFADRAPSGKAVLKNACPVQDASFLSKILFWWFSGLLFKGYRSPLQAEDLWSLREEDTSEKIISDLEEEWTAECTKLQQQENHLSTSIALGSRLPEQAQFLRKIQKEQSSGFCLLRTLARSFGPYFLTGTLCLMVHDAFMFSVPQVLSLLLGFMKDEDAPLWKGYFYASLMFLLSCLQSIFNHQYTYTCFTVGMRVKTAVMGLVYRKSLVVNSAARRTCTVGEIVNLVSADTQKLMDFVVYFNAVWLAPIEVTLCLFFLWQHLGPSALAGIATVIFIFPLNGFIARKRSKLQEIQMKYMDGRVKLMNEILNGIKILKFYAWEKAFLEQVLGYREKELKTLKKSQILYSVSIASFNSSSFLIAFAMFGVYVLIDDKNILDAQKIFVSMALINILKTPLSQLPFAMSTTMQALVSLKRLGKFLCQDELKPDNVTRESFKSGSRRLSVRLSVTEYMPFSRDLSQEQLISGDTNSITIEPLPDSDEDHIQEDLGKLTKADKARIGRVKLEMYIEYFHTIGLPLIVSIVFLYAFQQAASLSYNYWLSLWADQPVINGTQLNTDLKLGVYGALGFAQGIAIFGTTVAISLGCIIASRHLHLELLNNVLHSPMSFFETTPSGNLFNRFAKEIDAIDNMIPDGLKMMLGYFFKLMEVCIIVLMATPFAAVIILPMAFLYGFIQSFYVATSCQLRRLESVSRSPIYTHLNETVQGASVIRAFNEQSRFIMGVNHKVDQNQTAYFPRFVATRWLGVNLEFLGNGIVLAASILSVMAKGTLSPGMVGLAVSHSLQVTGFLSWIVRSWTDVENNIVSVERVKEYADTPKEAAWTIEGSSLPPSWPQKGTIEFHDYGLQYRKGLELALKGISVHIKEREKIGIVGRTGAGKSSLALGIFRILEAAKGQIFIDGINIAEIGLHDLRSRITINSNELYSQLA |
| CcpABCC6-2 | MQQQESALNGTQALGYKLSEQTQLLRKLHKEQISGFCLLRTLAKNFGPYFLTGTLCLVIQDAFMFSIPQVLSLLLGFVRDEDAPLWKGYLFAFLMFLLSCLQSLFNHQYMYTCFSVGMRVKTAVMGLVYRKSLVINSAARRTCTVGEIVNLVSADTQKLMDFVVYFNAVWVAPIEIALCLFFLWQRLGPSALAGIATVILIFPLNGFIAKMRSKLQEVQMKYMDGRIKLMNEILSGIKILKFYAWENAFRERVLGYREKELNALKKSQILYSISIASFNSSTFLIAFAMFGVYVLIDEKHVLDAQKVFVSMALINILKAPLSQLPFAMSTTMQTRILVTHGLSFLPQADLILVMEDGEITEMGSYAELLSRKNTFVQTLLKLFPCANARKVPPTELNMQIHLVLGTRKSVSRLSMTDFSIDLSQEQLISGDMGSASIQTMEAISDTEQEQDQEEVGRLTHADKAHTGRVKLEMYVEYFRTIGLAFIIPIIFLYAFQQAASLAYNYWLSLWADEPVVNGTQVDTDLKLGVFGALGFAQGIAIFGTTVAISLGGIIASRHLHLELLNNVLHSPMSFFESTPSGNLLNRFSKEIDAIDCMIPDGLKMMLGYVFKLLEVCIIVLMATPFAGVIILPLTLLYAFIQSFYVATSCQLRRLESVSRSPIYTHFNETVQGAGVIRAFGEQSRFILLANSRVDHNQTSYFPRFVATRWLAVNLEFLGNLLVLAAAILSVMGRATLSPGIVGLAVSHSLQAPWTIEDSPLPSDWPKSGSIGFQEYGLQYRRGLDWALKEISLSVNEREKVGIVGRTGAGKSSLALGIFRILEAAKGKIFIDGINIAEIGLHQLRSRITIIPQDPVLFSGSLRMNLDPFDGYNDEEVWRALELAHLKNFVSGLPDKLNHECSEGGENLRYKPLLSSVYSSILITSDRTVSDRIFWASVWVSGSWFAWPEPS |
| CcpABCC6-3 | MQQQESAMNGAQALGYKLSEQTQLLRKLHKEQSSGFCLLRTLAKNFGPYFLTGTLCLVIQDIFMFSIPQVLSRVYALIDSRGLIFQQDLAAAHSAKATSTWFKDHGIPVLNWPANSPDHNHIENLWSIVKRKMRYARPKNAEELKATIRAAWALITPEQCHRLIDSMPRRIAAIAFAMFGVYVLIDENHVLDAQKVFVSMALINILKAPLSQLPFAMSTTMQTRILVTHGLSFLPQADLILVIEDGEITEMGSYAELLSRKNTFADFAEAFSVSERKESATHRGTRKSVSRLSMTDFSIDLSQEQLISGDMGSASIQTMEAISDSEQEQDQEEVGRLTQADKAHTGRQAASLAYNYWLSLWADDPVVNGTQVDTDLKLGVFGALGFAQGIAIFGTTVAISLGGIIASRHLHLELVNNVLHSPMSFFESTPSGNLLNRFSKEIDSIDCMIPDGLKMMLGYVFKLLEVCIIVLMATPFAGVVILPLTMLYAFIQSFYVATSCQLRRLESVSRSPIYTHFNETVQGASVIRAFGEQSRFILLANSRVDHNQTSYFPRFVATRWLAVNLEFLGNLLVLAAAILSVMERSTLSPGIVGLAVSHSLQAPWTIEDSPLPSDWPRSGSIGFQEYGLQYRRGLDWALKEFPSV |
| CcpABCC7 | MQKSPVENASYLSKYFFWWTNPIMRKGFKEKLRPSVDKPIMRKGFKEKLRPSDVYQAPSKDAADILAERLEKEWDREVASGRKKPSLLRALARCFIQPFLLFGFLLYIGPAMFGLHHLGMQIRIALFSIIYKKAFLSHKMGPYKAQKVLLTNKRLALTSEIMENLHSVKAYGWEEIMETLIKNIRQDEVKLTRKIGSLRYFYSSAYFFSAIFVIVAAIVPHALSRGINLRRIFTTLSYCMVLRMTVTRQLPGSIQMWYDTMRLIWKIEEFLSKEEYRLMEYDLSITELELKDVTASWDEGPGELLERIKQENKANGQHNGDAGLFFTNLYVTPVLKDISLNLKKGEMLAVTGSMGSGKSSLLMTILGELVPSSGKIRHSGRISYSSQTAWIMPGTIRDNILFGLTYDEYRYKSVVKACQLEEDLAALPEKDKTPMAEGGLNLSGGQKARVALARAIYRDADVYLLDAPFTHLDIATEKEIFDKCLCKLMASKMRILVTNKIEHLKRADKILLLHNGESFFYGTFSELQSERPDFSSLLLGLEAYDNFSAERRCSILTETLHRVSVDESAGMRPERSAFRQVAPSMPVYIAERKSSVIVNTLGAARKASFIHIPEEEVRRTLPDRKFSLVPENELVDESFMGSDVYHNHGVHMAGQRRQSVLAFMTNAQGQGRRDQLQSSFRRRLSVVPQSELASELDIYTRRLSDSTYDISGVLEEENTEACLVDEIDEKEEVFETTKWNTYVRYVSNNKSLLYVLIFIFLIAAIEVAGSVAGIFLITDDLWKEEHQRVEPNMTKYSNMSSPGKNYAIIVTQTSSYYILYIYVATSESLLAMGFFRGLPFVHTMITISKKLHQKMLHAVLSAPMSVLNTMKTGRIMNRFTKDMATIDDMLPLLMFDFVQLTVVVVGCILVVSIVRPYIFLAATPLVIIFIVMRKYFLRTGQQLKQLETEARSPIFSHLIISLKGLWTIRAFERQAYFENLFHKTLNTHTATWFLYLSTLRWFLFRSDIIFVFFFTLTAWIAVGTNQDKPGEIGIVICLAMLILGTFQWCVATSIAVDGMMRSVDRVFKFIDLPSETPKLDKSKCSDLIIENVDAQADSSWPNRGQIDVQNLTVKYTEGGHAVLKDLSFTVEGRQRVGILGRTGSGKSSLFNALLKLVYTDGDISIDGVNWSKMPLQKWRKAFGVVPQKVFIFTGPFRMNLDPYGCHSDEELWRVTEEMMDKGQVKTYDSIQKLLNETSHLKQAISPAERLKLFPRRNSSMRTPQSKLSSVTQTLQEEAEDNIQDT |
| CcpABCC8 | MPLIKQRYRAVIEACSLEPDIDILPQGDQTEIGERIIAMKDGSIQTEGTLKDIQMSQPELFKQWKTLMHRQDKEFEKETVDENMTVLERKNLRRAMYSREALKTEVEEEEQSVESDEEDNLSRVMRLRATIPWRSCGTYLSSAGLLLLLLLLLSQLLKHSLLVAIDYWLAHWTSKVITAKIDAAAHNCSLVQDVGFSHSSYLLVFSLLCFLGIVLCLATSVAVEWTGLRVAKELHHNLLNNIILAPMRLFETTPLASILNRFSSDTNTIDQHIPATLECLSRSTLLCVSALGVISYVTPVFLIIVIPLAITCYFIQKYFRVASKDLQQLEDSTQLPLLSHFSETVEGLTTIRAFRYEPRFRQRLLEFTDANNMASLFLTAANRWLEVRMEYIGACIVLIAAVASITNSLYNHLSTGLVGLGLTYALMVSNYLNWMVRNLADMEVQLGAVRR |
| CcpABCC8-like | MNTVFTLSSVPQEKGPVPLKGSRWIWLALRQAFGRPLVLSITFRFIADLLGFAGPLCISGIVHHISSDNHSALPPRTFLQASYYVAIETGIKLRAAIQTKIYHKIMRLCTSNMSMGDMTTAQICSLVARDTNHLMWFFFLCPNLWAMPVQIVMGVLLLYSLLGVSALIGATVIVILAPLQYFVATRLSRAQKSTLEYTSERLKKTNELLRGIKLLKLYAWEHIFRSSVEETRRQELRNLRTFALYTSLSIFMNAAIPIAAVLTIFAVHVHVHSGTDFDLSPAVAFGSLSLFHILVTPLFLLSSVLHSTVKALVSVQKLSEFLGSTEIEIDQDPVVQTTATDNNNNNSNIKSVYALQLQAKLVNRKNKAKQDVNRNFEQQDQELTASANQDEDVCIKIVNGYFTWTQGCPTLSNIDIKVPFGQLTMIVGQVGCGKSSLLLAALGEMQKISGNITWNNLPKSDSEENSESPVYEWKTVSQIIAMRDGTVQTQGTLKDIQCAEPELFEQWRTLMNRQDKENTTELKRKSFRRPMYSTDTLATEDEDEDSTTSTDEDSLRAELRHRTTIPWSSFATYLRAAGLLLLPLLVLSQLTKHSLMVAIDFWLAHWTSRVIAAQIESAERNCTGTKDCEFSHSSYLQVFCGLCALGIVLCLVTSLAVEWT |
| CcpABCC9-1 | MGRNDYRFCWLSCLASRVGIVFLLLKCKTKLLFLCVCGWHGAAGFYRPPKAWRHGDMMSDDQLAMIYNKILRLSTSNMSMGEMTLSQINNLVAIETNQLMWFLFLCPNLWAMPVQIIMGVILLYYLLGDSALIGAGVILLLAPVQYLIATKLADTQKSTLDYSTDRLKKTTEILKGIKLLKLYAWENIFCDRVEETRGKELTSLKTFALYTSMSIFMNAAIPIAAVLATFVTHAYIEEVRLSPAKAFASLALFHILVTPLFLLSTVVRFAVKALVSVQKLSEFLQSDELGDDSWRNTEMSMSLEVGKKYKHHGDVQIFSMPILSDRIFAHSHCMIVTHVNEHRFACDFGHLSVISQNLSTKTINRKGRYRMDNYEQPMRRQLRPTETEDVAVQVNDGFFTWGSNLSTLSDINIRIPTGQLTMIVGQVGCGKSSLLLAMLGEMQTISGKVYWSNGHDNGLSHEELSKNRYSVAYAAQKSWLLNATVEENITFGSPFNKQSDHLMQEGILKFLQDDKRTVVLVTHKLQYLIHADWETEMESQTTLERKTLRRAFYSREAKSQTDDEDEEEEVEEDDDDNMSTTTSRRSKIPWKMCCRYLSSGGFLMVFLMVLSKLAKHSVMVAIDYWLAAWTSSNPHNQSLADPFINATNYTKNDDTQIAEVTHHCLLLFQMLSLILYIIVILKWSSLGVLQHRSYVPVFIILCGAAIALCLITSLTVEFLGVAAATNLHHNLLNKIIHAPIRFFDVTPLGQILNRFSADTNIIDQHIPQTLESLTRSTLLCLSAIGVIAFVTPAFLIALVPLMVAFYFIQKYFRVASKDLQDLDDSTQLPLLCYFSETAEGLTTIRAFRHEARFKQRMLELTDTNNTAYLFLSAANRWLEDYLGAVIVLTAAVAAIWTSSQSGLVGLGLTYALTVTNYLNWVVRNLSDLEVQMAAVKKVNSFLSTESENYEGSMDVSQVPKDWPQHGEIKIQDLCVRYDTMLKPVLKHINAYINPGQKVGICGRTGSGKSSLSLAFFNMVDVFEDSEHMGFV |
| CcpABCC9-2 | MSMCLEFGKKYKYHGDTKVINRKDRYRMDNYEQPMRRQLRPTETEDVAVEVNDGFFTWGSNLSTLTDINIRIPTGQLTMIVGQVGCGKSSLLLAMLGEMQTISGKVYWSKLPDYEIFFDGSISGHDTGQSHEERSKNRYSVAYAAQKSWLLNATVEENITFGSPFNKQSDHLMQEGILKFLQDDKRTVVLVTQEGILKFLQDDKRTVVLVTHKLQYLIHADWIIAMKDGSVLREGTLKDIQTHDVELYEHWKTLMNRQDQELEKETEMESQTTLERKTLRRAFYSREAKNHIDDEDEEEEVEEDDDDNMSTTTSRRSKIQWKMCCRYLSSGGFLMVFLMVSSKLAKHSVMVAIDYWLAAWTSSNPHNQSLADPFLNATNYTQNDDTQIAQVTCHCCFLFEMLSFILYIIVILKLSFLGVLQHRSCVPVFIILCGAAIALCLITSLTVEFLGVAAATNLHHNLLNKIIHAPIRFFDVTPLGQILNRFSADTNIIDQHIPPTLESLTRSTLLCLSAIGVIAFVTPTFLIALVPLAVAFYFIQKYFRVASKDLQDLDDSTQLPLLCHFSETAEGLTTIRAFRHEARFKQRMLELTDTNNTAYLFLSAANRWLEVRTDYLGAVIVLTAAVAAIWSTSGPDVDQSGLVGLGLTYALTVTNYLNWVVRNLADLEVQMAAVKKVNSFLSTESENYEGSMDISQVGICGRTGSGKSSLSLAFFNMVDVFEGKIVIDGIDICKLPLQTLRSRLSIILQDPVLFSGSI |
| CcpABCC10 | NGTISPCANQLLLGSLLHAVIAITSACYISVPRLTHIVSSLPIGWSFRATSTLLMALLFIGDLVLVFELSAPDVYLDVLADGCGVLAWLVHFGAVLALQRSMYRRTRGPALLPVLLMLSIPNLAFILTAYMHVIIHLGVSQPLQVVRLALTVTRAALVLVYLLGYIFPCYRTQRDLLSCNAEDVAPLIVSFEPEGGVTVAEDGCSWLSRILYLWLNPLLRRGKRGELERPCDVFQLPHRLRTKAVTLRFSQCWQKCLHPKGLERPERPDRLQRGNLQDNSWSEAEPDRMCEGGAQDDVKLLQVLHKAFGLRYYLLGVLKLAASMLAFAGPLLLGGLVNFMETDGAPLSKGVWCAVGLFASTFLAALLRNIFVYEVSKVALEARAAVISTIYSKALKVSASALARFNMGEVVNFMSTDTDRVVNFFNSFHEVWSLPFQFVLALYLLYLQVGVAFLGGVGVAVLLVPLNKVLASRILENNKHMLLHKDGRVKLMTEILFGIRVLKYYNWEEHFTQKIVEDPECNMGEVVNFMSTDTDRVVNFFNSFHEVWSLPFQFVLALYLLYLQVGVAFLGGVGVAVLLVPLNKVLASRILENNKHMLLHKDGRVKLMTEILFGIRVLKYYNWEEHFTQKIVEARKKELHHLKLLKYLDAVCVYTWAALPVVISILTFITYVLLGNSLTAAKVFSTLALVGMLILPLNAFPWVLNGTLEAKVSLDRIQRFLTIQDQDLTVYYSQVCPEDTLSAVEMNRASFSWKQSEEHDSDSVPDDKTEDGSPPHSFYLHALNLSVKRGSLVAVVGKVGCGKSSLLAAITGELSRFHAPYRGSETSGNMSSELKFYMTVYGSLAAANTVFTAARAFLFAYGAICAATVIHKRLLSSVLKATMTFFDTTPLGRILNRFSSDIYSVDDSLPFVLNILLANVFGLLGMLIVMSYGMPWVLLPLLPLGVLYFQTQCYYRHSSRELKRLCSLTLSPVYSHFSETLSGLSTVRASGHTTRFEEENERRLEQNQCCLFNSNAAMQWLDIRLQMIGVTVVTGISVIAVIQHQLKSIDPGLVGLSLSYALSITNLLSGLIFSFAQTEMQLVSIERIEEYSTNIPQEPQQASTE |
| CcpABCC12-1 | FQRLWDEEVAHVGLEKASLPAVVMRFQKTRFIVSFFVSVLFAFAAFVGPSILVHEILSYIEQPESSTLLHGIGLCVALFLSEFSKAFFASLLWAVNLRTAVRMKGAFSMLAFKKIISLRSLTSISIGETINVLTSDGYRMFEAITFGTFLLCVPFLLIICIIYACITLGYTALIGILVYLIFLPIQISIARLIGVFRRRAVSVTDRRVRTMNEVLTCIKLIKMYAWEESFEKTVTDIRKSEELLLQKAGYVQSLNTSFTTIVPTLATILTFIVHTALKWPLQPSSAYTIIAVFNCMRMSMGLLPFSVKSVAEGKVALTRLKKLMLVQNPKGYLTQDKNMDLALAMEKATFSWSLPDAMNATEKPQDPSESVTHQSDLKPSLRNISFTLSKGSLLGVCGNVGSGKTSLISSILEQMHLLSGSVSANGTLAYVSQQAWIFHGTYLEFCDEVVLLDNGEIKEAGTHCDLMKAKGRYAHLINNFQLEQSNENAESDSKTHTEHNDSKQSSPDKPNVNGIENPAFDLSDEKHVTNELPKDSTETKGKKDQLVTQEVSLEGSVTWRTYHQYCKAAGGYILLLFVILLFTLLVGSTAFSSWWLSYWLEQGSGNYTNSSSSGNISENPDLPFYQMIYGIIIVAMVLLSIAKGYTFTKVTLRASSKLHDTMFKRILGSPMSFFDTTPTGRLVNRFSKDQDEVDAVLPFNMENFLQFCLIVTFTILTICIVFPYLLIAVAVLTLIFATILYVFQRSIRQMKRMENVSRSPWISLTTSTIQGLSTIHAYDKRKQYIEQFKILSDTNSNHFMLFNCGTRWLSFWLDFLAATVTLIVALFVVLSSNEAINASLKGLALSYTIQ |
| CcpABCC12-2 | AYTIIAVFNCMRMSMGLLPFSVKAVAEGKVALTRLKAYTIIAVFNCMRMSMGLLPFSVKAVAEGKVALTRLKKLMLVQNPKGYLTQDKNMDLALAVEQASFSWSLPEAMNSTEKPKDPSENADFKPSLRNISFTLSKGSLLGVCGNVGSGKTSLISSILEQYLEFCDQVLLLDNGEIKEAGTHSELMKSKARYAHLINNFQLEQSNEDSESDSKTHTEHNDSKQTNTDEPKVNGIENPAFDMSDEKHNSSSNSSSSGNISENPDLPFYQMIYGIIIVVMVLLCIAKGYTFTKVTLRASSKLHDTMFKRILGSPMSFFDTTPTGRLVNRFSKDQDEVDAVLPFNMENFLQFCLIVTFTILTICVVFPYLLIAVGVLAIIFATILYVFQRSIRQMKRMENVSRSPWISLTTSTIQGLSTIHAYDKRKQIEQFKMLSDTNSNHFMLFNCGTRWLSFWLDFLSATVTLIVALFVVLSSNETISPSQKGLALSYTIQADSPSYHRILSSSSALSGDLSPFNSSTLLLSYSQLIAKLPEKLHSPVVILSIATLTPFTHYDKVSFQIAKLPEKLHSPVVENGENFSVGERQLMCMARALLRNSKIILLDEATASIDSETDSMIQHTIRDGFQHCTMLTIAHRINTVLESDRILVMDQGKVVEFDPPQDLIQRPNSLFASLLAAANQVNS |
| CcpABCC13 | MKEQEQSKGSLIHMNLFRINRVVVMGHRGLLQLCDLCKLYNEESVHTPSSVFEHEWEKQQNCNERLDLEEDSGRESLLVSRNTNTGYSLLYAIWVTFRPALVKVALLRLSTDIFSILVPLTLRWVILFSERQAVFDWAGYTYSLVVLGAVCCCAVSQHHFEKHSRIVAANAQAVLAGFLYRKRTQLIIMERSEQLIKEMLHKYQALKFLTWESWFQQRVTESRARELEILRILGYLTAFSMLNSICMPFLMHRGLLGKQIHRALRLKPKSKSIIDYNLRLLRPPLLELLGLFCVLKQKSFHLMASNSHVTVLPGHVLQKSFHLMASNSHVTGVKISTVILMRKVITIYPKNLAQDGILSVWTGEAKEVQGLEGWKELRNSRLSAYALLGLLQAFLVCCAAYCLTCASLRASHILHSKLLSDVLHMPMHSQKTDMRQLLQAFTQSRFYSAHSHIRHMGANPVASLECVTQCVEDPLSGPKHREVCLTHCLQALNQNLLVKYNKINTESRAALRLDGIAGIVLFLVTLILLETEPDSGLVVLALICAFNIKKAVHRYPQAASDINMDMLSMQNLCEFAKVEKEAAWKKSYRPPKDWPQYGEVKFRNYESEASSNGTPALRGINLTILKGEKIGVVSRGKTDTDALVSSLFRSVEARSGAVLIDDINIARMGLLDLRSRLQIISQVPVLFSGPLRANLDPFAQHTDAQVWLALELCHLKELVRLLPAQLLHPVQRTSLAYSFGQRRLLCLARALLARVRILLVEEALPGVDLETEDLVRQLIHTEFKHCTVLWLTQNPLTVMHTDRVLVLNKGQAVNVDAPSTLLQQGPLFSQ |
| CcpABCD1 | QRKSYRFGATCGSESSSGTAAVKQWSDNPEASHQVPLQSQEGSSSGSYEQGHRRSNSSETLLDRHGITAEDGGQRSGMPVRNGPYKSSEAIMDTTLKHAQRSSPERQVNGHAELARVHSAAGGRGTNGGVGYSEILMDYVWGKQQKMQAQQQQRLQVQSNAKTRLWADGASAPPPPYRNGFAQSQQLILGNCPPAYSPLMLRGNPGEPRRVKVSRTKSCGPFVPLQQHQQESILLSAYTETNTSNGTNATHNQQIDRLHRPPHYPLDSSAPMTPDDPTRSLHKALALEGLRDWYLRNALGQTANGGKGKEGMQTQRRRTTTSLHNSHPNPPLKHQPQSFQTDTSCPHIPQSATFHGHPLHGRSMELSLYQDTFSSKMQDMTLKESSNDAPTPGTLEFIETTSRRSHMICFEGVLDPFKSAASTADACEASCQWRSSLDGQIVKTIVKKDPRAFVVELTKWLLIATPATFVNSAILYLEGQLTLAFRTWLVTHAYMMYFSDQTYYRVSNMDGRLANPDQSLTEDVVMFAASVAHLYSNLTKPILDVVVTCYTLIKTAESKGANTTWSSIIAGIVVALTAKILRAFSPRFDTAGLEGYCRFGGFDVILLPLYLRVCVSACVFGCLRLCPAQIEMLQLQQSYTAVSKQINLILFKRLWYVMLEQFLMKYLWSASGLVMVAVPINTATRYSKYDSEDVKQAALVMKEEDLVSERTQAFTTARNLLNAAADAVERIMISYKEVTELAGYTARVYEMFEVFEDVRAGMYRRSLSELKPEETGAARKIKHGMRVEGPLQIRGNVIDVEQGIKCENLPIITPTGDVVVSSLNLQKWVIDGLPSSILKNVEKKDGASVIKLMTSLAVLRSNYWKKSDTSK |
| CcpABCD2 | MSNILHAASKVRWNQSTAAKRAVFLAAAAYGAKTLYPIICKQIQQRKTAKNNTSEPGHENGLISLHKATPETGSSLSKSPGVNAEFFKQILDLVKIVFPRFVTKELGLLCLHSVALVSRTFLSIYVAGLDGKIVKTIVEKQPRSFMIKLMKWLLIAIPATFVNSAIRYLECKLALAFRTRLVDHAYKTYFTNQTYYKVSNMDGRLANPDQSLTEDVMMFSQSIAHLYSNLTKPILDVILTSYTLIQTARDVMMFSQSIAHLYSNLTKPILDVILTSYTLIQTARSRGANATGPTLLAGLVVFATAKVLRACSPRFGKLVAEEAHRKGYLRYVHSRIIANAEEIAFYRGHTVEMRQLQKCYSTLAKQMNLILSKRLWYIMIEQFLMKYVWSASGLVMVAVPIITATGFADNELADGQTQVLVSERTEAFTTARNLLASGADAIERIMSSYKEVTELAGYTARVHNMFLVFDEVQRGIYKRSSAILTAEEMISGDDNRPEMHIDGPLEIKGKVIDVDKGIVCENVPIITPNGDVVVSSLNFKPVCWDAELDWKDVLSGGEKQRMGMARMFYHKPKYALLDECTSAVSIDVEGKIFQAAKDAGISLLSITHRPSLWKYHTHLLQFDGEGGWRFEQLDTATRLSLTEEKQRLESQLAGIPKMQLRLNELCKILGEDSVLKTVESKDED |
| CcpABCD3a-1 | MAAVSKYLTAKNSAVAGGILLVLYFLKQRRKSARLNRKKGSSNDLSSELKDGKKDRAAVDKLFFIRISRIIRIMVPRFFCKETWYLLLIAVMLVTRTYCDVWMIQNGTLIERGYTYYKMGNLDNRIANADQLLTQDVEKFCNSVVELYSNLSKGPATMMAYLLISGLFLTRLRRPIGKMTVTEQKYEGEYRYVNSRLITNSEEIAFYNGNIREKQTIHSTFKKLVDHLHNFIFFRFSMGMVDSIIAKYFATVVGYLVVSRPFLDLSHPRHLNSSHSELLEDYYQSGRMLLRMSQALGRIVLAGREMTRLSGFTTRITELMKVLNELNSGKYERTMVSLSEKDATEKLTLIPGSGRIINVDNIIKRTNDANVISHQVKSGANVLVCGPNGCGKSSLFRVLGERPYMTLGTLRDQVIYPDTHEDQKKKGISDQVLKEYLDNVQLGHILEREGSWDMVQDWMDVLSGGEKQRMAMARLFYHKPQFAILDECTSAVSVDVEDYIYNHCRKVGITLFTVSHRKSLWKHHKTFITTCQANEQASLKFVGIGDWGGLPIYPYYTPHEFDTANELGRIAKSSGLDFVLSLGDHFYFRGVRDVDDPRFKSTFESVFSHPALMAVPWYLVAGNHDHRGNISAQIAYSNRSERWIYPDLYYELNFKVPHSNTSITVLMIDTVVLCGNTYDRLEPVGPEDYAAAKQHLKWIEEKLQNTKSDFVIVAGHYPVWSIGHHGPTKCLVSKLRPLLKKYSVSLYLCGHDHSLQFIREDDGSSYVVSGTGVNAEIHTDHKNTFPPSWQLFSNAVNQTTGAFVYFEVNTSEMLINYIQEDGKCVYQTSVPKRKVQL |
| CcpABCD3a-2 | MAAVSKYLTVKNSAGAGGILLVLYFLQQRRRSAGLNRKKGSSNDLNSEKVGKKERAAVDKLFFIRISRILRVMVPRFFSKETWYLFLIAVMLVTRTYCDVWMIQNGTMIESAIIGRSTKGFKKYLFNFITAMPVIALVNNFLKLGLNELKLCFRVRLTKHLYDEYLKGYTYYKMGNLDNRIANADQLLTQDVEKFCNSVVDLYSNLSKPLLDIGLYIFKLTTAIGAQGPATMMAYLLISGLFLTRLRRPIGKMTVSEQKYEGEYRYVNSRLITNSEEIAFYNGNVREKQTIHSTFRKLVDHLHNFIFFRFSMGMVDSVIAKYFATVVGYLVVSRPFLDLSHPRHLTSSHAELLEDYYQSGRMLLRMSQALGRIVLAGREMTRLSGFTTRITELMKVLKELNSGKYERTMVSQSEKDGSEKLTLIPGSGRIINVDNIIKFDHTPLATPNGDILIRDLCFEVKSGANVLVCGPNGCGKSSLFRVLGELWPLFGGSLTKPERGKLFYVPQRPYMTLGSLRDQVIYPDTHEDQRKKGISDQVLKEYLDNVQLGHILEREGSWDMVQDWMDVLSGGEKQRMAMARLFYHKPQFAILDECTSAVSVDVEDYIYSHCRKVGITLFTVSHRKSLWKHHETFYTTCQANEQASLKFVGIGDWGGLPIYPYYTPHEFDTANELGRIAESSGLDFVLSLGDHFYFSGVRDVDDTRFKSTYESVFSHPALMAVPWYLVAGNHDHRGNISAQIAYSNRSERWIYPDLYYELNFKIPHSNTSVTVLMIDTVVLCGNTYDGLDPVGPENYAAAKQHLIWIEKKLQNTKSDFVIVAGHYPVWSIGHHGPTKCLISELRPLLKKYSVSLYLCGHDHSLQFIREDDGSSYVVSGTGVNADIFIDHKKSFPSSWQLFSNAVNQTTGAFVYFEVNTSEMLINYIQEDGKCVYQTSVPKRKVQLLTS |
| CcpABCD3b | MSYNRGFTYYQIGNLDNRIANPDQLITQDVEKFCDSVVDLYSNVSKPLLDILIYICKLNTAIGSLGPASLLSYLLFSGLLLTRLRRPIGKMTVMEQRYEGQYRYVNSRLITNRSVWDSISLHGCLSPEVFYEGCMISVYCFSEEIAFYNGNRREKQTINGTFQKLVDHLSRFIHFRFSMGVLDSIIAKYIAMAVGYLVISRPFLDLTNQRHVNSTYSERLEDYYQSGRMLISLAQALGRIVLAGREMNRLSGFTARITEIQEVLKELNSGRYERTMDYYQSGRMLISLAQALGRIVLAGREMNRLSGFTARITEIQEVLKELNSGRYERTMVTQRIKDDSVEKIPLIPGRGKIIIADNIIKFEHIPLVTPNGDILIRDLSFEVSSGTNVLVCGPNGCGKSSLFRVLGELWPLCGGQLTKPQRGKLFYVPQRPYMTLGSLRDQVIYPDTHEDQKKKGTSDLVLKEYLDNVQLGHILEREGSWDIVQDWMDVLSGGEKQRMAMARLFYHKPQFAILDECTSAVSVDVEDFIYSHCRKMARLFYHKPQFAILDECTSAVSVDVEDFIYSHCRKVGITLFTVSHRKSLWKHHEYYLHMDGRGNYKFKPITKETVEFGS |
| CcpABCD4-1 | MPYNSAHHCVVEVENFLLLLGGEDQWNPNGKHSTNFVSRYDPRFNSWVQLPPMQERRASFFACCLDKHLYVVGGRNETGGVHNGEYVSWLYCYDPVMDVWARKQDMNTKRAIHALAGMNDRLYAIGGNHLKGNSYSCNPNLSSSGGTYCVRNSNQNCFFCDSIPSNTYTPLQIQLVIYQVGLIPSQFYEVLSEKNYGKFKNLVLFAVMLILINSTLKSLDQYISSLLYVSWRKSLTEELHRTYFKGRVYYTLNVLCKDIDNPDQRISQDVERLCKQISTMASRLLISPFTVTYYTYQCFNSAGWIGFVSIFGYFVAGTIINKILIGPIVSMLVEQEKLEGDFRFKHMQIRVNAESAAFYRAVGVNTFDYLGSILSYIVIAIPIFAGDYDGLTPGELSALVSKNAFVCIYLINCFTQLIDLSTTVSDVAGYTHRIEELREVMADIAKKQCDQDQYDPLSKDEPYSDRELQSVPGDTAFVLDCLSYKSPVSVELLVKDLTLKISQGTHMLVVGNTGTGKTSLLRVLNGLWEPCSGSVEMTTCFGPRGVLFLPQRAYLTDGTLREQVIYPLKDVYPSSGMMFYRQEKCRGFVLLDCFTCSPNLQKARMEQNLFGGAPRFLTRPKAFSLCVGRDASLSCTIVGNPVPVVTWEKDKMLLSAAGRFKTVEDGDVYRLTIYDLTLEDSGQYMCRAKNNVGEAYAAVTLKVGLPETVIDRAPVFTVKPVSTRVGLGGDVTFYCRVAAHPAPNFDWEKDGRYLGETNRIKIISENESSSLRIQSVRSLDSGTYTCRAQNSIGRAHAAAALVVDLQDTHLLNADKSSSLLSHMQKRKEDMRKDISLYRTMETSSTTHTASSSMITEELGSLGLAYDQKQRVSALTSMLPKGVFTRTCMLTEGKHAKLSCFVTGHPKPQIIWRKDGANISEGRRHVMYEDQAENFILKVLYCKQSDNGLYTCNASNLAGQTYSAVLVIVKGKNNVCRYLLLATRRHVMYEDQAENFILKVLYCKQSDNGLYTCNASNLAGQTYSAVLVIVKEPKIPFKPEPKIPFKKKLQDVEVKEKETATLQCEVPMPNTKASWFMEETHLEESAKYRMDVEGTLRRLTIHHVTTNDDAVYICEMKEGSRTVAELTVLGNITKKLPRRTVVPVSDTVIFCVELEKPVDDAYWTRNGERLKEDSRIIIARINRQYTLTIRECTAEDSGEVAFIAHDCKTSTRFSVTAPRKHPPDPPVEPVVRNKTDSSITLCWSPPDSERPVPITGYIVERRKVGVQTWVKVTSITVSSTEYTISEIPEEASYQFRISAVNDFGQSAYLEVPGTFYLEPTASVKAGLVNCSAHVGEEATFTVELSTVCSGSWTINDRMIRSGSEYLITRSKTTHTLVIREVSMELNGAQVKFVGGGSQSGATLSVKAAPARFTNKSSQAEVFTFSMHSSAQMYTEVSDSSVQVVWIKNGKELRMGKKYEATSMERKRILTVHNVDHEDVGIYECMCDGDKMSVQLALKEEPTKFLNKPRAQLQQNAALVGDVVLSCEVASPGTAVVWKKEQIEIMEDKRTTFISQGTQRKLVIKGAKQSDEGHYSCETAEDKMTFLVKIK |
| CcpABCD4-2 | MQAMKNSGVRRDQRISQDVERLCKQMSTMASRLLISPFTVTYYTYQCFNSAGWIGFVSIFGYFVVGSILNKILIGPIVSMLVEQEKLEGDFRFKHMQIRVNAEAAAFYRAGKVEHMRTDRRLQMLLSTQRSLMNKELWLYIGVNTFDYLGSILSYIVIAIPIFAGDYDGLTPGELSALVSKNAFVCIYLINCFTQLIDLSTTVSDVAGYTHRIGELREVMADIAKKQCEQDQYDPLSKDEPYSVPADTAFVLDRLSYKSPVSEELLVKDLTLKISQGTHMLVVGNTGTGKTSLLRVLNGLWEACNGSVEMTTCFGPRGVLFLPQKAYLTDGTLREQVIYPLKDIYPSSGSIDDERILKYLELVGLSNLLSRIGGLDTKVDWNWYDVLSPGEMQRLCFARLFYLQPKYAVLDEATSALTEEAEGQLYKACKQLGMTLISLGHRSTLKKKARMDQNLFGGAPRFLTRPKAFSLCVGRDASLSCTIVGNPVPVVTWEKDKMLLSAGGRFKKVEDGDVYRLTIYDLTLEDSGQYMCRAKNNVGEAYAAVTLKVGLPETVIDRAPVFTVKPVSTRVGLGGEVTFYCRVAAHPAPNFDWEKDGRYLGETNRIKIISEKDSSSMRIQSVRSLDSGTYTCRAQNSIGRAQAAASLVVDLQDTRLLNADKSTSLLSHMQKRKEEMRKDISLYRTKESSSTKLSTSSSTITEELGSLGLPYDQEQRVSALTSMLPKGVFTRTCMVTEGKHAKLSCFVTGHPKPQIIWRKDGANISEGRRHVMYEDQAENFILKVLYCKQCDNGLYTCNASNLAGQTYSAVLVFILKVLYCKQCDNGLYTCNASNLAGQTYSAVLVIVKEPKIPFKKKLQDVEVKEKETATLQCEVPVPNTKASWFMEETRLEENTKYRMDVEGTLRRLTIHNVTTNDDAVYICEMKEGSRTVAELTVLGNITKKLPRRTVVPVSDTVIFCVELEKPVDDAYWTRNGERLKEDSRIIIARINRQYTLTIRECTAEDSGEVAFIAHDCKTSTRFSVTAPRKHPPDPPLEPVVRNKTDSSITLCWSPPDSERPVPITGYIVERRKVGAQTWVKVTSTSVSSTEYTISEISEEASYQFRISAVNDFGQSAYLEVPGTFYLEPTASVKTGLVNCSAHVGEEATFTVELSAVCSGSWTINDRMIRSGSEYLITRSKTTHTLVIREVSMELNGAQVKFVGGGSQSVSTLSVKAAPARFTNKSSQVEVFTFSMHSSAQMYTEVSDSSIQVVWMKNGKELRMGKKYEATSVERKRTLTVHNVDREDVGIYECMCDGDKMSVQLALKGETSCFSH |
| CcpABCD4-like | MALIRRSLGLCPQHDVLFDNLTVREHLLFFTQLKGYPRKKIPATRWDLNSGAGYHMVIVKDAFCNVSEITRLVHMYVPDATLESSAGAELSYILPKESTSRFELLFAELEMNRDELGIASYGASVTTMEEVFLRVGKLVDSSLDIQAIQLPALQYQHERRVGKLVDSSLDIQAIQLPALQYQHERRSHDWTMDDSSSISGMTDVTDFTDSGTMISEDGSNIKLNTGTRLYMQQFYAMFLKRALYSWRNWKVMVAQFLVPLVFTVLALVVARTLPGSQITPLLRLALKHYGPTHVPVAVDVNAGPLATALAEIYTAQLPSQNAIAATNITDLSEYVLYNAVREGGAFNEHCVVGATFRSGSRKNTEVIGYFNNQGYHTPATALMLVDNALYKLLAGPNASIQTGNNPMPRNISETAQSQLSEGQTGFAIAINLMYGMASLASTFALLLVSERSVKSKHVQQVSGVYLSNFWFSALLWDLINFLLPCLLMLLVFQVFSVEAFVAENHLVDVLLLLLLYGWAVIPLMYLLSFLFSTAATAYTRLTIFNILSGTATFLAVTIMTIPELKLEDMSHLLDKIFLIFPNYCLGMSFSEFYQNYEIITFCTSSDFADFICKSYNITYQVNYFSMDEPGVGRFLVAMSLQGVVFIALLFLIELRCVHILLNLCRRRKKVLLLAEEALQPEDRDVAEERKRVMDCQPVVESMVDSPLILQELCKVFGVLETAKEKYSIEDYCVSQISLEQ |
| CcpABCE1-1 | MADRNTRIAIVNHDKCKPKKCRQECKKSCPVVRMGKLCIEVTPQSKIVWISESLCIGCGICIKSVASLSADRMWNLYQVVLMVLCSDWSDLLHLRERNVEDLSGGELQRFACAVVCIQRADIFMFDEPSSYLDVKQRLRAAITIRSLISPDRYIIVVEHDLSVLDYLSDFICCLYGVPSAYGVVTMPFSVREGINIFLDGFVPTENLRFRETSLVFKVAETANEEEVKKMCRYQYPNMKKCMGEFSLTITEGEFTDSEIMVMLGENGTGKTTFIRMLAGGLKPDEGGEVPILNVSYKPQKISPKFKGSVRALLHDKIRDAYTHPQFVTDVMKPMQIESIIDQDLKDEEALCSFRGEVPILNVSYKPQKISPKFKGSVRALLHDKIRDAYTHPQFVTDVMKPMQIESIIDQDDVEQKKSGNYFFLDD |
| CcpABCE1-2 | MARESQRKATCILHSLLRVDQAEDHPKVLRGHLETGETLTGVGWMFAGWRLLLVDARGSGSTGSRVKICVAAVRHQTSQMADKNTRIAIVRATSNQPNGRQEHQNCHREPRQVQAQEMSSGVQEELSGGKLCIEVTPQSKIVWISESLCIGCGICIKELVCEQLDLLHLRERNVEDLSGGELQRFACAVVCIQRADIFMFDEPSSYLDVKQRLRAAITIRSLISPDRYIIVVEHDLSVLDYLSDFICCLYGVPSAYGVVTMPFSVREGINIFLDGYVPTENLRFRETSLVFKVAETAAEEEVKKMCRYQYPNMKKSMGEFTLGITEGEFTDSEIMVMLGENGTGKTTFIRMLAGGLKPDGGGEVPILNVSYKPQKISPKFKGSVRALLHEKIRDAYTHPQFVTDVMKPMQIESIIDQDPLVRFNKETRCIL |
| CcpABCF1-like | MAVPLFALLAVCVDVSAVMFIGAIQLSPSFINNTFTLLWAGGLLRTCLLLFISFTYPGSPAWMRGFEGVQTAVIHGLLYPVYISFIWACDRSTVELVWGWHTCQGGYAVLALSLLLWKRYVPTLLPTAKKQKTEKKGSASLQRLLGYMRPFRGRFAVVFFFVVISSLGEMAIPHYTGKMTDWIMNEDEPEAFNHAITVMTLMTIMSAVCEFVCDLIYNITMSRIHTSIQGLVFQSVLKQEIGFFDKASTGDIVSRITTDTNTMSESLSEKLSLLMWYFMRVIFLFGSMLLLSTRLSIFTALGLPIIWIIPEFSGRFYQKLSVQVQESLAKANDVATETFSSMKTVRSFANEDGETERYRKCLEDTYALNKVEAAAYAASTWTNSMSSLALKVSILYYGGRLVTGSDVSSGDLVSFVLYELQFTSAVEMQFIVGISSVGVGLSVLGGMEQSSGKRIRKPSLLYEGFESPSLPHVPPPGPPPLQQPPVTDPSRQGRTTNQLQFLHKVLVKALWRHHFAWPFHEPVDAVRLNLPDYHKIIKQPMDMGSIKKRLENNYYRSASECLHDFNTMFTNCYIYNKPTDDIVLMAQSLEKVFLQKVAQMPQEEIELPPPTPKSRGAKTFKGHRGKGGGSVTSAHQVPAVSQSAYSPSSPDTPDSQFSIPPQTLLSNSGPPPPLMTPPPTQPTAKKKGVKRKADTTTPTTLSFPVTAAARMGGMGKGHGSAGEMPHSLSSVPVDCSPSMGVNEPTHLQPILGRPLSRRPIKPPRKDLPDSVRPHQRRGKLSKQLRYCSGVLKELLCKKHAAYAWPFYKPVDASTLGLHDYHDIIKHPMDLSTIKRKMDEREYRDASSSALMSDSCSPTATSTTTDHDVVWLWHANCRNSKKSSSSRFTMPAAVSLPHYDSEEEEDGLPMSYDDKRQLSLDINKLPGEKLGRVVHIIQSREPSLRDTNPEEIEIDFETLKPSTLRELERYVMTCLRKNAPQSIR |
| CcpABCF2a | MQWHRTATSQETDGFQVKRPGDVNVRCTLLLMLDYQPPQFKLDPRLARLLGIHTQTRSCIIQALWQYVKTNKLQDSHEKEYINCDKYFQQIFDGPRLKFSEIPQRLTNLLLPPDPIVINHVISVDPNDQKKTACYDIDVEVDDPLKSQMNGFLLSTANHQEIASLDNKIHETIESINQLKIQRDFMLSFSRDPKGYIQDWICSQNRDLKLMTDTVGNPEEERRAEFYNQPWSQEAVSRYFYCKSKRKTAKRKKQPKLVNVQRKPEDGVNGDAEKPETQQNGAAEDNGVASLTKELDEFELKKTEARAVTGVLASHPNSTDVHISSLSLTFHGQELLSDTSLELNSGRRYGLIGLNGTGKSMLLSAIGHREVPIPEHIDIYHLTREMAPSEKTALECVMEGNYDQYIKTREELEENQMKRFNWEQDQIAHMKTMSLPAAPPSYAEATAGDKEQGTVDHESASSPPSYAEATAGDKEQGTGPGYSNAYAADMSSPFSDPSSSSSFDGLSGSNWEDKNVRRMFIRKVFCILMVQLMVTFGVVSLFTFCEPVRKFVQYNRVFYLTSYMTFMGTYLMLVCSTNARRRYPTNMILLAIFTLAMSYMAGMLASYHNTKVVMMCVGITALVCLAITLFCFQTRVDFTSCHGLLFSLMMVLMVTGLLLFFTAPFGYIPWLHTAYAGFGALVFTLFLAFDMQLLIGNRRYSLNPEEHVFGAICLYMDVVYIFLFFLQLFGSPFSLFGSSDERTEEQKLEDELILLLKKAK |
| CcpABCF2-2 | MEFYLSYYHKIRELVPESQAYMDLLAFERKLDQTIMRKRVDIQEALKRPMKQKRKLRLYISNTFNPAKPDAEDSEGSIASWELRVEGKLLDDPGKQKRKFSSFFKSLVIELDKDLYGPDNHLVEWHRTPTTQETDGFQVKRPGDVNVRCTLLLMLDYQPPQFKLDPRLARLLGIHTQTRSCIIQALWQYVKTNKLQDPHEKEYINCDKYFQQIFDCPRLKFCEIPQRLTNLLLPPDPIVINHVSANQQEIASLDNKIHETIESINQLKIQRDFMLSFSRDPKGYIQDWICSQNRDLKLMTDTVGNPEEERRAAFYNQPWSQEAVSRYFYCKFFDAHEVQIHCVSSHLERFQRCLFLVFTVKMPSDLAKKKAAKKKEAAKARQRTKKPEDGVNGDAEKPEIQQNGAAEVNGVASLTKELDEFELKKTEARAVTGVLASHPNSTDVHISSLSLTFHGQELLSDTSLELNSGRRYGLIGLNGTGKSMLLSAIGHREVPIPEHIDIYHLTREMAPSEKTALECVMEVDEERIQLEREAERLAHEDYFLNGVCTNIIHLHQRKLKYYTVKRLILSPGNYDQYVKTREELEENQMKRFNWEQDQIAHMKNYIARFGHGSAKLIKEKEEMRKIIGRYVLCGSQYLVFTFKIPIKIMEPGGLWTMSLPAAPPSYAEAIAGDKEQGAGSFTYASQPPPMPPPTIPMHPSWAYVHPGPMQDVCLGPGYSNVYAADMSSPFSDPSSSRSFVQDVCLGPGYSNVYAADMSSPFSDPSSSSSFDGLSGNNWEDKNVRRMFIRKVFCILMVQLMVTFGVVSLFTFCEPVRKFVQYNRVFYLTSYMTFMGTYLMLVCSTNARRRYPTNMILLAIFTLAMSYMAGMLASYHNTKVVMMCVGITALVCLAVTLFCFQSRVDFTTCHGLLFSLMMVLMITGLLLFFTAPFGYIPWLHTAYAGFGALVFTLFLAFDMQLLIGNRRYSLNPEEHVFGAICLYMDVVYIFLFSFQLFGSQILNAALGPTVQDVCLGPGYSNVYAADMSSPFSDPSSSSSFDGLSGNNWEDKNVRRMFIRKV |
| CcpABCF3 | VLDSGGSDFEDGEEVFDAIGDVLQEVGDKNEDDIRKICFQMFNTLQLTKCDSGQQQVLLEAPVQLSQVSADAASTANDVHGIWMLKRNPNTTVDAKKLEKAEAKLKAKHERRNEKDSQKSSSSPLVLEEASASQASSKKENRVDSSGKNRSYDIRIENFDVSFGESVSDVSALSASTANDVHGIWMLKRNPNTTVDAKKLEKAEAKLKAKHERRNEKDSQKSSSSPLVLEEASASQASSKKENRVDSSGKNRSYDIRIENFDVSFGERADGLESVRLSEIYAKLEEIEADKAPARASVILAGLGFSAIMQQQTTKEFSGGWRMRLALARALFARPDLLLLDGEENVFIDRFRYNANRAAQVQSKLKLLEKLPELKPLVKESEAVLRFPDNFEKLSPPILQLDEVEFGYNPDQRIFNGLSVSADLESRICIGGVVLVSHDERLIRMVCKELWVCEAGRVHRVEGGFDEYKDILQEQFRREGYL |
| CcpABCG1 | MEFYCYTGAETREEGGMRCRRSLAAGVNPQCHRAGCSSLAPWTLEAVGWSELPGTDELAASRPQCGGAAAVREGTEESAPFARWPEPAAIRRNGEEQGTGDSCRLPESGGAIAVHWAAEEYRAVHRGRSRERGTPAGCPKVEEPSPSTGRRRSIVPSTEGHPVPPPGTAEEFTQLTFVLFEQALIRTTRYFNKNVSRYQLISNSRKRMKADVKCGLDSSSCFQVVSLMKALAQGGRTVICTIHQPSAKVFELFDKLYVLSQGQCIYRGRVSSLIPYLRELGLNCPTYHNPADFIMEVASGEYGDQTAWLVKAVQECKCEKDYKSETNGNGVQNPFLWHRPSDEVLTHLRIMSHLGIGILIGLLYLGIGNEAKKVLSNSGFLFFTMLFLMFAALMPTVLTFPLEMGVFLREHLNYWYSLKAYYLAKTMADIPFQVATFVGPVTAIPVLLFSGFFVSFDTIPKIPPMDIIHFLCQVATFVGPVTAIPVLLFSGFFVSFDTIPKYLQWISYISYVRYGFEGVILSIYGLDREDLHCDKDETCHFQKSEAILKELDVEDAKLYMDFIVLGIFFVSLRLIAYFVLRYKISAER |
| CcpABCG2 | MADTDVLIDEDLDYNVSRTPVSTRMSFSSSRRGVTVSFHNINYSVKMKSGFMCKRKVTQKNILIELKVTLANTSHTVPEWYYEAGTERHIRSNWEREIIVRAVCLFLDVLAARKDPAGLSGELLIDGAPQPPNFKCLSGYVVQDDVVMGTLTVRENLRFSAALRLPKSIRQRVKDEKIEKLIQELGLSKVADSRVLALVFIRKLLDFVFSKRDLSWLDDLMPESKKKKWKMHSKRYHHPHHTLLLVAACNSVIGQSPMTNHCRQCFLRTGHQHVFYSHFQENQCVLMEDEGIVQVQENQCVLMEDEGIVQVPLEGHFKCWFPKAHSDGTHSLLRILCSTVEVAIGHKRGDEEQAADLDRETSLNRVCHIRHVFKVHLELVIKLKYVNGG |
| CcpABCG2b | MSAYVVQDDILMGTLTVRENLLFSGNLRLPRKQCSTADKEKKVESIIQELGLEDCADTKKSLAELYRESHYCAAVKEELKCITGRSDPSSEAKTKAASYATSFFYQLKVVCWRTMLNVVRNPQTSYAQMAMNIICALLIGLIYYQMPLSLPEALQNRHENSGGYYRTSVYFLSKVFVDLLPNRIVPIFIFSSICYYMMGLNPSFTAFLCFALTMSMVSLAGVSLAFLVSASVSTFAMANVLIALPFVFMMVFGGFLVNLNSMLNWLSWLKWASIFKYGLDAVTINEMKGQVFYSVNATLTGEMYLQSQGIDYSLWGFWQNQVALLGIVLVCMTFAYIQLRRINRWK |
| CcpABCG2c | MLDELVVKEQPSTDMEESIPCFQAPGPTLTFHHLRYHIRERLGMFSREWVEKDILKDVSGIMNPGMNAIMGPTGSGKTSLLDVIAGRKDPKGLKSGQVLVDNTTVTSDLRLCSAYVVQALQKGQFDHLTLMNKGEIIYAGAANKAITYFEDLGYKCEPFNNPADFFLDVTNGTILPQIHNNKSEKCSSSEEMEENENPLAVIYRQSPYFLSVKDRLNQISDGLDPEVTKGDRVSYATPFYYQLMLVSGRTVRNILRNPQTSYAQLFLNIFFGILVGLIYYQIPHTLPEALQNRTGAFFFLVINMVFGNLSAVELFISERVLFIHENSSGFYRTSVYFLSKVFADLIPNRILPVFIFSAIPYFMMGLKPDVEAFFLYCLTMSMVSLSAVSLAFLVSASVGSFAMANILIAMPYVFMMVFGGFLVNLNSMLSWMSWLKWASIFRYGYNALAINELKGQVFISNYTSSLRGDVYLDHQEIDHSTWGFWQNQVALTGIMCVCLILAYVQLCRINRWK |
| CcpABCG2d | MTDNAVFEHDQITEETGKNGMSGSNVIAMEHIKKQHGATVSFHSIRYRVEQKSGSICRRTTVHKEILVDLNFLDVLAVRKDPAGLSGEVLINGALQPPNFKCLSGYVVQVQCMITLLRYFEFEVAAVSQQSIEDQLVEKYKNCSFARSLKAELERITRAKDNNIKAKCHTITYNSSFFHQLRWVLWRTFWNLMLNPQTSVAQLAVTTLMAAIVGAIFYGVKDNQSGIQNRFGVLFFITTNQCFSTLSAAELFITERKLFIHEYTSGYYRVSVYFLSKILSDIITQRTVPTILFTCVVYFMIGLKPTVAAFFIFMLTVILVSCAAVSMTMAISADQSVIFSGLLVNLKSIMDWLSWLKYLSIPRYGLVALEINEFVGLKFCGNVSAVMSSPAADILMCTGEQHLTFQGIDYSSWGLWHNHIALTLMTLIFLIIAYIKLRFIPKFS |
| CcpABCG2-like | MSLAFCGNDNNSAAYNVDAGVLNNGCFLDALTVVPHVFLLFITFPILFIGECQRQYPHITNIERGATGRSNGRGKRLGDQSSKVHIHHSTWLHFPGHNLRWILTFILLFIIVCEIAEGIVSDGFNQSVHLHLYMPSCLAFMAAITSIIFYHNIETSNFPKLLMTLLIYWVLAFVSKTIKFVKYTEHGIGLRQLRFGITGLLTLLYGLLLAVEINVILTRRYMCFANPTEVKPPEDLQDLGVRFLQPFVNLLSKSTYWWMNTFITAAHRRPIDLKVIGKLPIAMRALTNYLKLREAFEAQKWGEGLLHLCAHGSKARVTCAVTCLCAQLQRPQDTQLQGPKWIWTALRQAFGRPLFLSITYRFMADLLGFAGPLCISGIVHHLSKENHTIQPPVKHLGIYFISSQEFLANAYVLAVLLFFALLLQRTFLQASYYVAIETGINLRGAIQTKIFNKIMRLCTSNMSMGGMTVGQICNLVAIDTNQLMWFFFLCPNLFAMPVQIIVGVILLYYLLGISALIGATVITLLAPVQYFVATKLSDAQKSTLEYSSERLKKTNELLRGIKLLKLYAWEHIFCSSVEETRGKELTSLQTFALYTSISSFLPIGMPVCEFDLVKDPEDPEVQDFRRNILNVCKDSVELRDASGSHSRALYVYPPNVESSSELPKHIYGKLDKGQIIVVIWVIVSPNNDKQKYTLKINHDCVPEHVIAEAIRKKTRSMLLSAEQLKMCVQEYQGKYILKVCGCDEYLLEKYPISQYKYVRSCIMLSRLPNLMLMSKDSLYSQLPMDNFTMPSYARRISTATPYMNGEASTKSLWTINSTLRIRVLCATYVNVNIRDIDKIYVRTGIYHGGEQLCDNVNTQRVPCSNPRWNEWLTYDMYIPDIPRAARLCLSICSVKGRRGQKR |
| CcpABCG4 | VCYRGRVEINEKDIDGTCSSTCPDAKTYAVPQNHKCSRIENCRTKESGWKHWFTRCNYCDCDCFTPCELKALGMAAKSLEPGSVSIPMEETKRAGGAPPDAAMLTHLKKVENHITEAQRFSHLPQRSAVDLEFTELSYTIREGPCWRRRELERLCKKCRIAWDDFCAVVKRSAKPSMADPNSLIGETGDSRKGTLSGSLISVRFSHKFHSSTTKLAIDLPMASAVVFVAHRPRSIALHISSKTTVSGPDSSMAWRSLAWKTWSMAMECRAEACPAKEYARPARAEVVLHCIRWQWSFSMCMSSAVSLLAVIEVASGEYGDLNPVLFEAVQGGLCSDDGKKNSSDKNDATTSCPSQCYNESGYIEKHTFATSTLTQFCILFKRTFITICRDMVLTHLRVMSHLCIGVLIGLLYLNIGNDASKVFNNTGFLFFSMLFLMFAALMPTVLTFPLEMSVFIREHLNYWYSLKAYYLAKTMADIPFQVICPIMYCSIVYWMTEQPPEAGRYVLFMALSTSTALVAQSLGLLIGAASTSLQVATFVGPVSAIPVLLFSGFFVNFDTIPKYLQWSSYVSYVRYGFEGVILSIYGMNRSELECPGLVCKFQKPEEVLQLLDVEDAKLYVDFIVLGVFFFILRLATYLVLRYKVKSER |
| CcpABCG4b | MSEKTNLQDADGNVSRVIRVGTRKSQLARIQTDSVVEKLKELHPDVHFEIVAMSTTGDKILDTALSKIGEKSLFTKELENALEKNEVDLVVHSLKDLPTVLPVGFTIGSVLKRENPHDAVVLHPKHKGKRLDSLANKRLQIFGMAVKASDLGSVSITVEESLCNGGTQPQEAPLLTHLKKVENHITEAQRFSHLPKRSAVDLEFRDLSYTIREGPWWKKQAVYLSQGQCIYKGSVPYLIPYLRGLGLHCPTYHNPADFIIEVASGEYGDLNPVLFEAVQGGMCALEHKCNCIDKTTLTPCTAKCVKDSGHVESHTFATSSLTQFCILFKRTFITICRDQVLTHLRLMSHISIGVLIGLLYLNIGNDASKVFNNTGFLFFSMLFLMFGALMPTVLTFPLEMAVFLREHLNYWYSLKAYYLAKTMADIPFQVLCPIMYCSIVYWMTEQPPEASRYLLFLALSICTALVAQSLGLLVGAASTSLQVATFVGPVTAIPVLLFSGFFVNFDTIPEYLQWSSYVSYVRYGFEGVILSIYGMNRTELECPGRVCKFQRPEEVLQLLDVEDAKLYMDFIVLGIFFLILRLATYLVLRYKIKSER |
| CcpABCG5 | MHEDTPTGLCWNCKRCPPCLIPGCRMSPLFAAEQLRQIGDVGIHTGLGYENRLAAALKPDINLKAKRRSRDNKRSRAACIQVKNSRNDRLLPHLTVRETLAFVAKLRLPAHFTQKQRDQRVDDVIAELRLRQCAHTRVGNDYVRGVSGGERRRVSIAVQLLWNPGEVLPYRLLNTLFHVKVEKNTEDFMWKSEDCGSLALDAPQRRQVFNDYRDLVTLVVHGLEALLMSLLIGFLYFGAGDQGLSVQDTVALLYMIGALTPFAVVLDVIAKCHSERAMLYHELEDGMYSVTSYFFAKVLGELPEHCAFTLVYGVPIYWLAGLNSAPDRFLLNFLLVWMMVYCSRCMALFVAALPTLQTSSFMGNALFTVFYLTAGFVISLENMWLGKSIIEHFISD |
| CcpABCG8-1 | MSVQSAFHSEDSFGSTNDKQNRGQDILFSSPEEDSSLYFTYSGGRNEVEVRNLNYERRTASLLFLLYSGGRNEVEVRNLNYEVDMAAQTPWYERLSELKMPWEMHGNKQTVIKDLNLCVHSGQMLAVIGSSGILILDEPTSGLDSFTAHNLVITLYRLARGNRLVLLSVHQPRSDIFQLFDLVVLLSSGSAVYCGQAKDMVAYFTSLGYPCPRYCNPSDYYVDLISIDRRSPEKEAQCLEKARMLAAQFVEKVKNTEDFMWKSEDCGSPALDAPQRRQVIQREPCFIMNWKMACTQSTGHSERAMLYHELEDGMYSVTSYFFAKLLTLMRYFKLQCLFQVLGELPEHCAFTLVYGVPIYWLAGLNSAPERFLLNFLLVWLTVYCSRCMALFVAAALPTLQTSSFLGNSLFTVFYLTAGFVISLENMWLVASWFSYISFMRWGFEGMLQVQFRGTRIPITIGNLSVEFDGIKVVEMMKMNQYPLYSCYLVPIAVAFVSSCSIICHSNSLNRSPVRIELLKNNGIPLKRLIGFYSAICIWLRTNPLVFSTSQSDSPELPDRGENMSTSL |
| CcpABCG8-2 | ILILDEPTSGLDSFTAHNLVITLYRLARGNRLVLLSVHQPRSDIFQLFDLVVLLSSGSAVYCGQAKDMVAYFTSLGYPCPRYCNPSDYYVDLISIDRRSPEKEAQCLEKARMLAAQFVEKLGMRGLSIQDTVALLYMIGALTPFAVVLDVIAKCHSERAMLYHELEDGMYSVTSYFFAKLLTLMRYFKLQCLFQVLGELPEHCAFTLVYGVPIYWLAGLNSAPLSICILSHLVS |
