# Supplementary material for: Genome-Wide Identification, Characterization and Phylogenetic Analysis of ATP-Binding Cassette (ABC) Transporter Genes in Common Carp (Cyprinus carpio)
Source: PLoS One. 2016 Apr 8;11(4):e0153246. doi: 10.1371/journal.pone.0153246 (PMC4825979; doi:10.1371/journal.pone.0153246)
Supplement: S2 Table — (DOCX) [file pone.0153246.s002.docx]

**S2 Table. All common carp ABC gene primer sequence.**

| **Gene name** | **Primer sequence** |
| --- | --- |
| ABCA1a-1(F) | CCTGAGAGCGTTCAGGACAT |
| ABCA1a-1(R) | CGTAGGCCTCTTGCTGGAAA |
| ABCA1a-2(F) | GGAGAGTGGATGGTTCCAGA |
| ABCA1a-2(R) | CCACATACGCTGTACTGGGA |
| ABCA1b-1(F) | CAGTGATGATGCTCCACAGGA |
| ABCA1b-1(R) | CCAAACTGACGGGGGTCTTT |
| ABCA1b-2(F) | GATCAAGAGAGCGAGGCCG |
| ABCA1b-2(R) | CAGGGATTGAGAGCAAGGCT |
| ABCA2(F) | GCAAACTGGACCCTAAACAGC |
| ABCA2(R) | CATGTCGCACCAACTCCAGA |
| ABCA3b(F) | CTGGAAGCAAAATCACGCCC |
| ABCA3b(R) | TGACGCCATGCCATACATGA |
| ABCA4a(F) | CTGGATTTGGGACTCGCTGT |
| ABCA4a(R) | AGCTGCATTCTGGATGGCTT |
| ABCA4b(F) | GAGACAATGAGGCTGCGAGT |
| ABCA4b(R) | TCGACCTCAGGAGAAACTGG |
| ABCA5-1(F) | GCTGGCGTTTACACCCTTTG |
| ABCA5-1(R) | TTCACTGGGGTTCCGTTGAC |
| ABCA5-2(F) | TGGACCAAGTGTTACCAGGC |
| ABCA5-2(R) | AGGAAAACCTGAGTGGGTGT |
| ABCA12(F) | GTGCGTGGGTTCACTTTGAC |
| ABCA12(R) | TTCTGTTCGCTGGCACATCT |
| ABCB4(F) | ATCGGCTGGTTTGATGTCAATG |
| ABCB4(R) | AGGACTGTCAGGAAGGAGATG |
| ABCB5-1(F) | GCCCTCACGAACTCTTCAGT |
| ABCB5-1(R) | GCTCCCTGACCCAAAGAGAA |
| ABCB5-2(F) | ACGGACAGAAGAAGGCAGTG |
| ABCB5-2(R) | GGACCCACCGAGACACATTT |
| ABCB9(F) | GCAAATGGCGAACAGCAGTC |
| ABCB9(R) | AACCAAACGGTCCTCAATGC |
| ABCB11a(F) | GCGTGGGGAATTAGAAGGGA |
| ABCB11a(R) | GGCATCAGTGGCTAGTCTGG |
| ABCB11b(F) | TCGCAGCTGTCAAACCTTGT |
| ABCB11b(R) | TCGTCACTTGAACAGACGGC |
| ABCC1(F) | TTCTGCCATCCGAGAGAGTG |
| ABCC1(R) | GTCTTCTTTGAAGCCGCAACAT |
| ABCC2-1(F) | AGCGGACGCCCAAAGATTTA |
| ABCC2-1(R) | AAATACAGCGAAGGTCGCCA |
| ABCC2-2(F) | ACATACTGCGCTTCCCACTC |
| ABCC2-2(R) | CTCCAGGTGACGAAACACCA |
| ABCC4-1(F) | ATTCGCAGCACTGGTTAATGA |
| ABCC4-1(R) | GTCTCCTCGGCCATGGTAT |
| ABCC4-2(F) | ATTCGCAGCGCTGGTTAATG |
| ABCC4-2(R) | GATCGCTGCAGCTCTGAGTA |
| ABCC5-1(F) | ACTGGTGCCAGTTTGGCTT |
| ABCC5-1(R) | TGGTCTCAAGCAGCAAGCAC |
| ABCC5-2(F) | CGTTGGGATTTTGGGTCTGT |
| ABCC5-2(R) | TCATGCCTCCTCTTGTCTGC |
| ABCC6a(F) | ATCTGCGCTAGCAGGCAT |
| ABCC6a(R) | GTTCCAAGTGAAGATGGCGG |
| ABCC6-2(F) | GCCTGGGAGAATGCCTTTAG |
| ABCC6-2(R) | AAATGGCCACAGTAGTGCCAA |
| ABCC6-3(F) | AGGAAAGTGCCACCCATAGA |
| ABCC6-3(R) | GACTGTCCTCGATTGTCCAT |
| ABCC7(F) | CGTGTCTCGGTGGATGAGAG |
| ABCC7(R) | TCTGGGTCACAATGATGGCA |
| ABCC8(F) | GCCATGTACTCCAGAGAGGC |
| ABCC8(R) | AAGTGATGGCCAGCGGTAT |
| ABCC8-like(F) | AAACTCCGAGCAGCCATTCA |
| ABCC8-like(R) | CGTCCATGTGAAGTAGCCGT |
| ABCC9-1(F) | CTGCTGAACGCCACTGTAGA |
| ABCC9-1(R) | CCTCAGCGATCTGTGTGTCA |
| ABCC9-2(F) | AGCCATGAAGGACGGTTCAG |
| ABCC9-2(R) | CCTGAGCGATCTGTGTGTCA |
| ABCC10(F) | AGGCCTTTGGTCTACGCTAC |
| ABCC10(R) | TTTTTACGGGCCTCCACAA |
| ABCC12-1(F) | CCGCTGTGAGAATGAAGGGA |
| ABCC12-1(R) | TAGCTGACACAGACCCACTC |
| ABCC12-2(F) | CACTTTGCGTGCTTCTTCCA |
| ABCC12-2(R) | GTGGTATGACGGAGAGTCGG |
| ABCC13(F) | ATGGCATCCTGAGTGTGTGG |
| ABCC13(R) | CAGCAATGCCATCTAACCGC |
| ABCD1(F) | CACGACCTCTCTGCACAACT |
| ABCD1(R) | GAGCGTGTAACAGGTCACCA |
| ABCD2(F) | ATCGTGTTCCCGAGGTTTGT |
| ABCD2(R) | TCACTCACCAACACTTGCGT |
| ABCD3a-1(F) | CTCACCCTCGACACCTGAAC |
| ABCD3a-1(R) | CATGCGGTGTGTAGTAGGGG |
| ABCD3a-2(F) | GAGGTGAAATCCGGAGCCAA |
| ABCD3a-2(R) | CGTGTCAAACTCATGCGGTG |
| ABCD3b(F) | CAACCTGGACAACCGTATTGC |
| ABCD3b(R) | AGAGGGATTTTCTCTACTGAATCAT |
| ABCD4-1(F) | TTTTGGACCGAGAGGAGTGC |
| ABCD4-1(R) | CGAGTCCCAAGCTTCCAAGT |
| ABCD4-2(F) | ACCTGGAACTCGTTGGTTTGT |
| ABCD4-2(R) | TCCGCATTTCTTCTTTCCGC |
| ABCD4-like(F) | CATGACGGATGTGACGGAT |
| ABCD4-like(R) | TGGGGTTATTTCCCGTCTGG |
| ABCE1-1(F) | TGCCGATCGGATGTGGAAT |
| ABCE1-1(R) | CCTTCCGTGATGGTAAGCGA |
| ABCE1-2(F) | GGTGTAGGCTGGATGTTTGC |
| ABCE1-2(R) | CACCTCCTCCGTCTGGTTTG |
| ABCF1-like(F) | GAGCCGAATACACACGTCCA |
| ABCF1-like(R) | GCCCATGTCCATCGGTTGTT |
| ABCF2a(F) | CAGAGGATGGCGTGAATGGT |
| ABCF2a(R) | TTGTCCTCCCAGTTGCTTCC |
| ABCF2-2(F) | CTCTAGAGACCCCAAGGGC |
| ABCF2-2(R) | GTGCTATCTGGTCCTGCTCC |
| ABCF3(F) | AGCGGCGGCTCAGATTTC |
| ABCF3(R) | GCTAACCTCATCCTCCAGCC |
| ABCG1(F) | TGAGGGAACGGAGGAGTCAG |
| ABCG1(R) | AGGACTTTCTTGGCCTCGTT |
| ABCG2(F) | GTGCAAGAGGAAGGTTACGC |
| ABCG2(R) | TTTCTTCTTGCTCTCGGGCAT |
| ABCG2b(F) | TGAAGCCAAGACCAAGGCAG |
| ABCG2b(R) | ACTGGCACTGACAAGGAAGG |
| ABCG2c(F) | CGCTACCACATCAGAGAACGA |
| ABCG2c(R) | TGTTCTGAAGGGCCTCTGGT |
| ABCG2d(F) | CTGGGTGTTGTGGAGAACCT |
| ABCG2d(R) | ATCACGGCAGAGACATTCCC |
| ABCG2-like(F) | TGCAGTTACGTGTTTGTGCG |
| ABCG2-like(R) | GCATCTCGCAGCTCTACTGA |
| ABCG4(F) | TGGTATGGCTGCGAAATCCT |
| ABCG4(R) | ATAACACTGGGAGGGACACG |
| ABCG4b(F) | TCCGTGTTGGCACCAGAAAA |
| ABCG4b(R) | GGCGCAGATGTGTCAAAACC |
| ABCG5(F) | GGGACAACAAGAGGAGCAGA |
| ABCG5(R) | CATGTACAGCAAAGCCACCG |
| ABCG8-1(F) | ACCCTTGTCCACGATACTGC |
| ABCG8-1(R) | TGAGACGCTTTAGAGGGATGC |
| ABCG8-2(F) | TCTCTTGGGTACCCTTGTCCA |
| ABCG8-2(R) | GGACCTGAAAGAGACACTGGAG |
